# An Immature Myeloid/Myeloid-Suppressor Cell Response Associated with Necrotizing Inflammation Mediates Lethal Pulmonary Tularemia

**DOI:** 10.1371/journal.ppat.1005517

**Published:** 2016-03-25

**Authors:** Sivakumar Periasamy, Dorina Avram, Amanda McCabe, Katherine C. MacNamara, Timothy J. Sellati, Jonathan A. Harton

**Affiliations:** Center for Immunology and Microbial Disease, Albany Medical College, Albany, New York, United States of America; Stanford University School of Medicine, UNITED STATES

## Abstract

Inhalation of *Francisella tularensis* (Ft) causes acute and fatal pneumonia. The lung cytokine milieu favors exponential Ft replication, but the mechanisms underlying acute pathogenesis and death remain unknown. Evaluation of the sequential and systemic host immune response in pulmonary tularemia reveals that in contrast to overwhelming bacterial burden or cytokine production, an overt innate cellular response to Ft drives tissue pathology and host mortality. Lethal infection with Ft elicits medullary and extra-medullary myelopoiesis supporting recruitment of large numbers of immature myeloid cells and MDSC to the lungs. These cells fail to mature and die, leading to subsequent necrotic lung damage, loss of pulmonary function, and host death that is partially dependent upon immature Ly6G+ cells. Acceleration of this process may account for the rapid lethality seen with Ft SchuS4. In contrast, during sub-lethal infection with Ft LVS the pulmonary cellular response is characterized by a predominance of mature neutrophils and monocytes required for protection, suggesting a required threshold for lethal bacterial infection. Further, eliciting a mature phagocyte response provides transient, but dramatic, innate protection against Ft SchuS4. This study reveals that the nature of the myeloid cell response may be the primary determinant of host mortality versus survival following Francisella infection.

## Author Summary

*Francisella tularensis* (Ft) causes an acute fatal pneumonia upon inhalation of the bacteria. Natural infection, usually from contact with infected rabbits, is rare, but a low infectious dose of Ft and easy aerosolization has prompted its use as a biological weapon. During infection Ft appears to evade host defenses by various means, but how disease develops and leads to death of infected individuals remains unknown. Work to date suggests that a failure to control bacteria, delayed cytokines, endotoxic shock, suppression of immunity, or a combination of these is responsible for fatal disease. We have evaluated the sequence of systemic host immune responses and found that an inappropriate response of mostly immature, ineffective, and dying phagocytic cells likely explains the tissue damage and death accompanying Ft pneumonia. Promoting a more appropriate phagocyte response decreases susceptibility to lethal Ft infection and favors survival of the host.

## Introduction

*Francisella tularensis* (Ft) is a highly pathogenic gram-negative bacterium classified as a category ‘A’ biothreat agent by the CDC [1]. A virulent strain (SchuS4) of Ft subsp. *tularensis* (Type A) is highly pathogenic to humans and animals, while the less virulent live vaccine strain (LVS) of Ft subsp. *holartica* (Type B), is non-pathogenic to humans [1]. Unlike non-fatal skin infection, inhalation of as few as 10 cfu of SchuS4 results in acute pulmonary tularemia with high mortality in mice, while lethal LVS infection requires higher bacterial numbers.

Ft evades host defense through various mechanisms including, subversion of bacterial recognition by host cells, phagolysosomal escape and ROS scavenging (reviewed in [2]). Ft initially replicates in host cells without eliciting inflammatory cytokines such as TNFα, IL-1β and IL-6 [3–6]. Ft also elicits an anti-inflammatory lung milieu, thought to contribute to tularemia severity [3, 6]. Consequently, unfettered exponential Ft replication results in overwhelming bacterial burden that account for acute death in SchuS4 infection [7]. Inflammatory cytokines manifest in lungs later (>3 dpi), but are too late to prevent death [8]. Multiple cytokines and HMGB-1 elaborated in later days, however, suggest bacterial sepsis-associated death [4, 5].

Despite delayed cytokine responses, Ft elicits acute lung infiltration by neutrophils/poly-morphonuclear cells (PMN) and macrophages (MΦ) [6, 9–11], but their pathogenic role and mechanism of failure to control Ft are not clear. PMN are important in controlling Ft as depletion of PMN increased LVS susceptibility and bacterial burden in mice [12–14]. In contrast, *MMP9* ^-/-^ mice, which are largely deficient in lung PMN recruitment, exhibited protection and reduced bacterial burden during LVS infection [11], suggesting a deleterious role for excessive PMN. Also, a protective or detrimental outcome in tularemia may depend upon the extent of PMN recruitment [15].

Development of regulatory or tolerogenic myeloid cells in lungs and spleen may limit host control of Ft infection and early production of regulatory cytokines and eicanosoids (e.g., TGFβ, IL-10 and PGE_2_) has been reported in pulmonary tularemia [3, 6, 16–18]. These regulatory cytokines might favor the development of suppressive cells such as myeloid-derived suppressor cells (MDSC). MDSC are a heterogeneous population of mature and immature myeloid cells (IMC) with the capacity to inhibit innate and adaptive immunity [19]. MDSC play a critical role in a number of microbial infections [20–22]. Specifically, MDSC alter early innate as well as Th1 and CD8+ T cell responses during acute and sepsis-like infections [23–26]. In acute infections, MyD88-dependent G-CSF induction results in emergency myelopoesis and depletion of BM cellularity leading to expansion of MDSC in spleen/LN or infection target organs. In addition, cytokines like IL-6, IL-1, and S100 or TNF activate MDSC at the infection site, where MDSC are noted for T-cell inhibition and defects in phagocytosis and, at least for G-MDSC, accelerated apoptosis [23–31]. Thus, acute infiltration of myeloid cells during pulmonary tularemia might favor development of MDSC-like cells in lungs and spleen. Although IMC accumulation in the spleen following intradermal Ft infection is reported [17], the phenotypic and functional characteristics of lung infiltrating myeloid cells in pulmonary tularemia are unknown. Thus, it is unclear whether unfettered Ft replication, lack or abundance of cytokines, immature/immunosuppressive cells, some combination of these or other mechanisms are ultimately responsible for pathogenesis and host mortality in pulmonary tularemia.

Here, we sought to better understand the pathogenic mechanism leading to acute death in lethal pulmonary tularemia. An overt innate cellular response to Ft, but not overwhelming bacterial burden or cytokines, results in host death. Ft induces medullary and extra-medullary myelopoiesis contributing to infiltration of lungs by immature myeloid cells (IMC) and mature myeloid cells that are phenotypically and functionally similar to MDSC. While IMC/MDSC contribute to the immunosuppressive nature of pulmonary tularemia and their depletion partially ameliorates mortality following LVS infection, the death of infiltrating myeloid cells is temporally associated with necrotic tissue damage and host death. Further, adoptive transfer of Ly6G^+^ and Ly6C^+^ IMC/MDSC enhances mice mortality following Ft LVS infection. In contrast, preferential recruitment of mature phagocytes protects against lethal SchuS4 infection.

## Results

### Host responses mediate acute death in pulmonary tularemia

**Fig 1:**
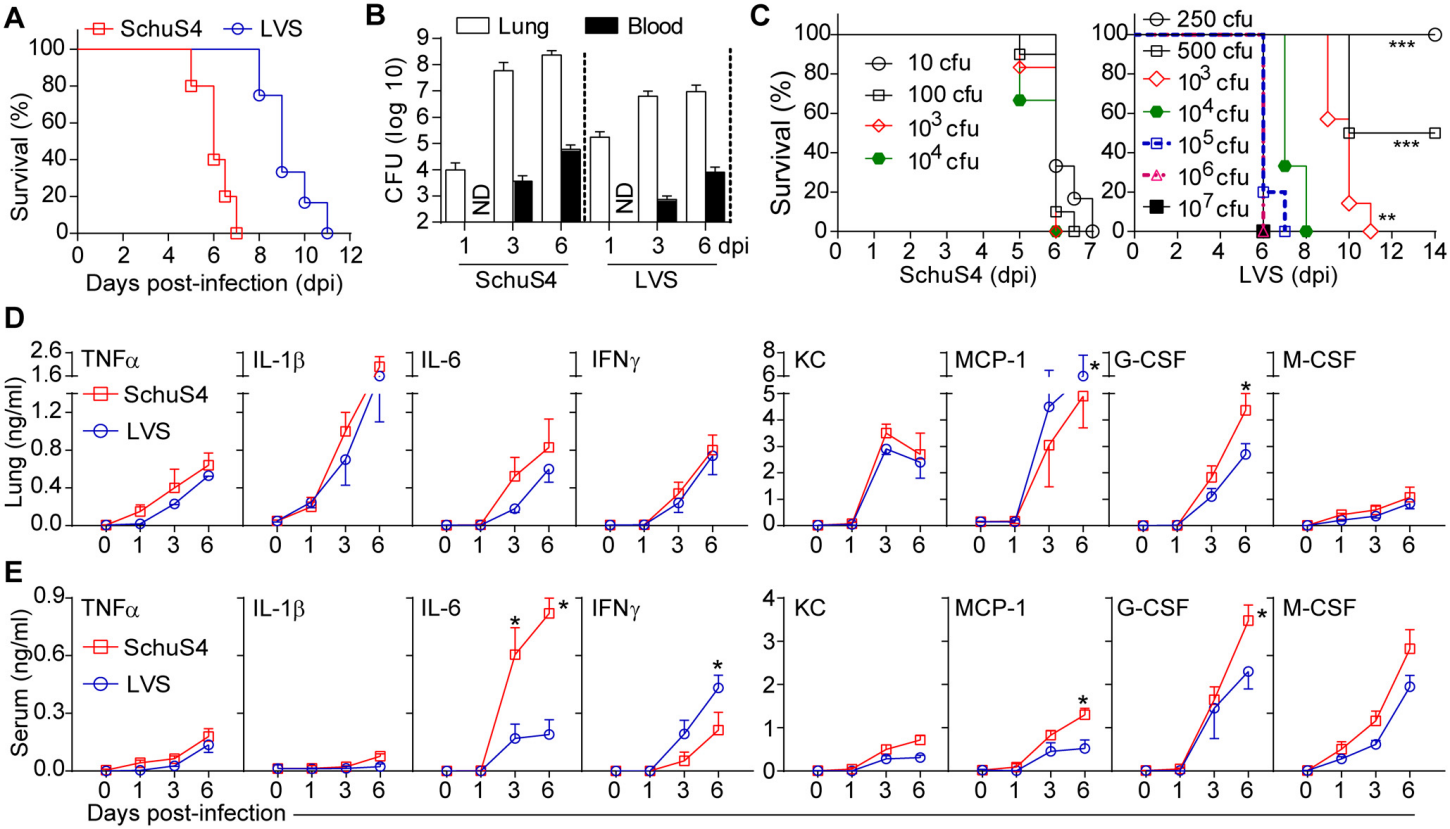
Mortality, bacterial burden, and cytokine kinetics in pulmonary tularemia. (A) Survival of Ft SchuS4 (10 cfu) or Ft LVS (1000 cfu) infected C57BL/6 mice (% survival of three independent experiments). (B) Lung and blood bacterial burden in Ft infection (mean ± SD of three independent experiments; the bacterial burden at all days post-infection (dpi) was significantly (p<0.001) different from the initial inoculum of 10 cfu (SchuS4) or 1000 cfu (LVS) at 0 dpi, ANOVA with Tukey’s post-test). (C) Survival with escalating Ft numbers (mean % survival of two independent experiments, Log-rank (Mantel-Cox) test, **p<0.01 and ***p<0.001 indicate difference from 10^5^−10^7^ cfu groups). (D) Cytokine and chemokine levels in lungs and (E) serum (mean ± SD of two (SchuS4, n = 6 mice) or three (LVS, n = 9 mice) independent experiments, Student’s t-test, *p<0.05 represents differences between SchuS4 and LVS strains at indicated dpi. In panels B, D and E, for SchuS4 infection at 6 dpi, ~50% of the mice had died and only the remaining live mice (n = 6) were used for analysis. As previous studies have demonstrated that most of these cytokines are significantly induced at or after 3 dpi, statistics for differences in cytokine induction at various dpi following SchuS4 or LVS infection are unmarked.

Despite intensified research in the last decade, complete understanding of the mechanisms of acute pathogenesis and high mortality in pulmonary tularemia remains elusive. C57BL/6 wild-type mice infected intranasally with a minimum lethal dose of Ft SchuS4 (10 cfu) or LVS (1000 cfu) are clinically normal until 3 days post-infection (dpi), but exhibit clinical signs of acute infection (e.g. anorexia, ruffled fur, huddling, dyspnea and hunched posture) from 5 dpi onwards. At these doses, death occurs at 5–7 dpi (SchuS4) or at 8–11 dpi (LVS) (Fig 1A). Male and female mice are equally susceptible (S1A Fig). Lethal infection is accompanied by exponential replication of Ft in lungs and dissemination to blood and vital organs (Figs 1B and S1B), with tens of millions of bacteria in tissues, consistent with the prevalent hypothesis that overwhelming bacterial load accounts for sudden death. In contrast, even with 10^4^ SchuS4 or 10^7^ LVS, death occurs no sooner than 5–6 dpi (Fig 1C). Further, infection with 10^7^ LVS resulted in bacterial burdens exceeding those in mice receiving a minimally lethal dose (S1C Fig), therefore the seemingly obligate time window preceding death does not reflect a spatial limitation for replicating Ft. Thus, while exponential Ft replication establishes a systemic infection, some host-mediated processes, not overwhelming bacterial burden, are likely responsible for death.

Comprehensive cytokine/chemokine analysis reveals a host response to Ft largely similar between LVS and SchuS4 with significant levels of many factors (e.g., TNFα, IL-1β and IL-6) appearing at 3 dpi onwards during infection (Fig 1D and 1E). Early suppression of these inflammatory cytokines by Ft through inhibition of NF-kB or other signaling pathways is suggested to facilitate a lethal infection [3, 32–33]. In contrast, we have demonstrated that Ft activates NF-kB, similar to other TLR agonists [6]. Consistently, the directly or indirectly NF-kB regulated cytokines (e.g., TGFβ and IL-12p70) and eicosanoids (e.g., LTB_4_ and PGE_2_) are induced early (<3 dpi) in infection (Figs 1D, 1E and S1D). Together with G-CSF production (Fig 1D and 1E), this early response is consistent with recruitment of myeloid cells which serve as a replicative niche [34]. IL-1β, TNFα, IL-6, and IFNγ were substantially induced in lung (Fig 1D) later (>3 dpi) during Ft infection. However, there was little induction of IL-2 or IL-4 at any time point (S1D Fig). During SchuS4 infection, serum levels of IL-6 and G-CSF were higher while IFNγ levels were reduced. However, with the exception of IL-6, serum cytokine kinetics were similar for SchuS4 and LVS infection, but of lower magnitude in comparison to lungs (Fig 1E). IL-1β, TNFα and IL-6 mediate endotoxic death as deficiency or blockade of these cytokines is protective [35, 36]. However, *TNF* ^-/-^, *IL-6* ^-/-^ and *Casp1/11* ^-/-^ mice are more susceptible to pulmonary tularemia [37, 38] and (S1E Fig), demonstrating the essential role of these cytokines in tularemia. IL-12 is also a mediator of endotoxic shock, but the limiting quantity of IL-12p70 precludes its functional ability, despite the presence of IL-12p40 at 3 and 6 dpi (S1D Fig). Of note, Ft LPS is largely inert and fails to elicit endotoxemic death in mice or MΦ cytokine response, when compared to an equivalent dose of *E*. *coli* LPS [39] and (S1F–S1H Fig). Also, inactivated Ft (iFt) at 2 x 10^7^ (i.e., equivalent to bacterial burden at 3 dpi) does not elicit any of these cytokine responses or mortality (S1I and S1J Fig). Although soluble mediators are likely important in protection, an overwhelming host cellular response likely mediates death in pulmonary tularemia.

### Overt inflammation is pathogenic and detrimental in pulmonary tularemia

**Fig 2:**
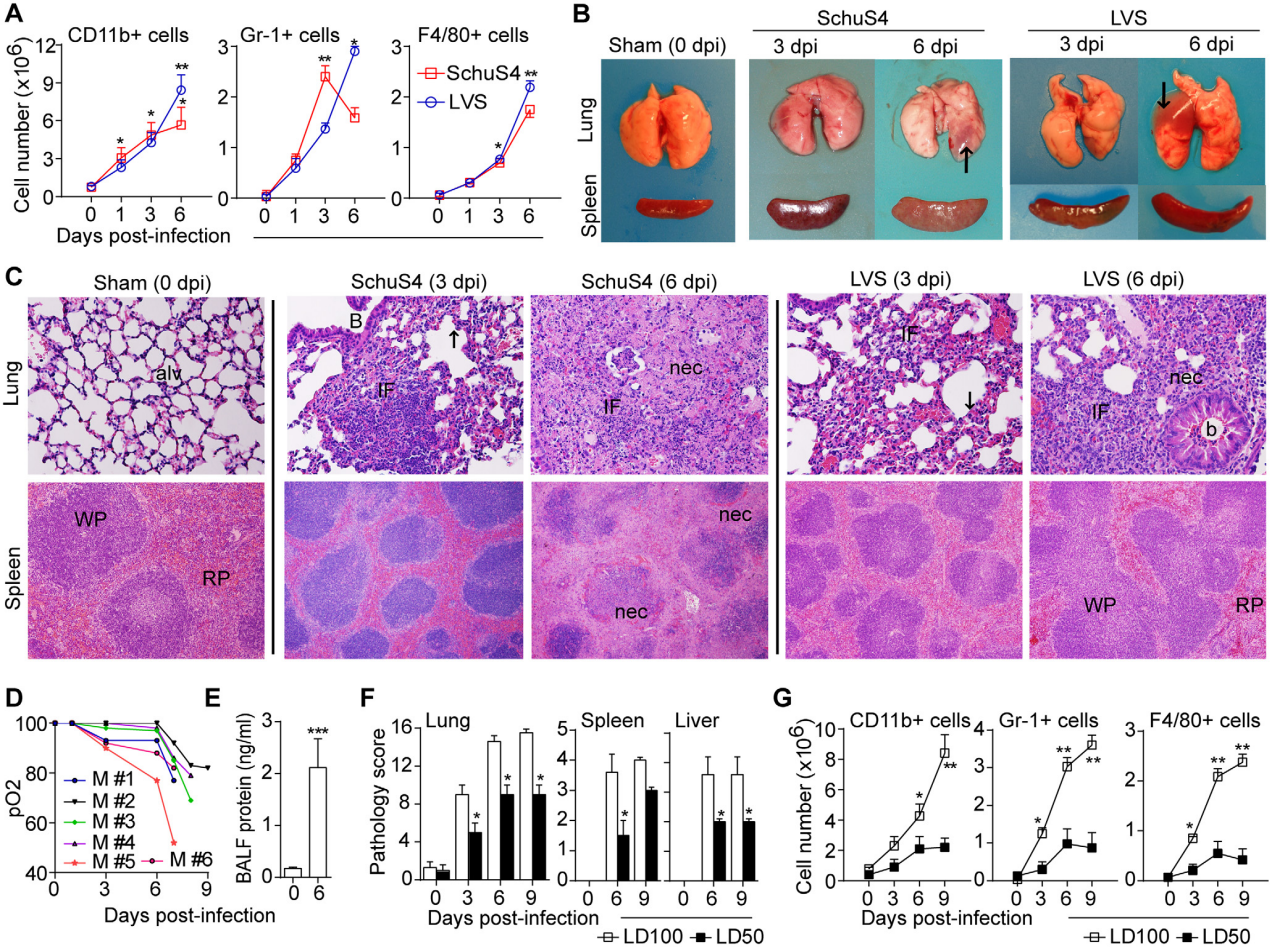
Overt inflammation is pathogenic and detrimental. (A) Total cell numbers of innate myeloid cells in lungs following LVS (1000 cfu) or SchuS4 (10 cfu) infection (mean ± SD of two (SchuS4, n = 6 mice) or three (LVS, n = 9 mice) independent experiments, Students t-test, *p<0.05, **p<0.01 for differences compared to uninfected mice at 0 dpi). (B) Gross pathology of lungs (arrows indicate consolidation) and spleen. (C) Histology of lung and spleen infected with SchuS4 or LVS, as shown in panel A. For lung (top): alv-alveolar wall, b-bronchiole, IF-inflammatory foci, and nec-necrosis. Note: hyaline membrane-like changes in alveolar epithelium (arrow) at 3 dpi indicating acute-lung injury, and necrotizing inflammation at 6 dpi (HE, 400x). For spleen (middle): WP-white pulp, RP-red pulp (He, 200x). For liver (bottom): granulomatous (arrow, 3 dpi) or necrotizing (arrow, 6 dpi) inflammation (HE, 200x). Microscopic images are representative of two independent experiments (n = 6 mice). (D) Oxygen saturation (SpO2) levels in individual LVS-infected (1000 cfu) mice through 9 dpi (all mice died by day 10) with a mean pO2 of 73.3 ± 11.6 of the last measurements prior to death). (E) Protein level in BAL fluid from control or LVS-infected (1000 cfu) mice at 6 dpi (mean ± SD of two experiments, Student’s t-test, *** p<0.001). (F) Comparative pathology scores for lungs, spleen and liver of lethal (1000 cfu) vs sub-lethal (500 cfu) Ft LVS infection. Pathology score was calculated by analysis of section of whole lung (n = 6 mice) for location/type/and extent of inflammation and necrosis (see Methods) (mean ± SD of two experiments, Mann-Whitney test, *p<0.05). (G) Myeloid cell number in lungs for lethal (1000 cfu) vs sub-lethal (500 cfu) Ft LVS infection (mean ± SD of two independent experiments (n = 8 mice), Students t-test, *p<0.05, **p<0.01).

Given the elevated levels of chemokines and eicosanoids, we investigated the kinetics of immune cell recruitment in tissues. In lethal pulmonary tularemia, progressive infiltration of CD11b^+^ myeloid cells, including Gr-1^+^PMN and F4/80^+^MΦ, was noticed in lungs (Figs 2A and S2A) and spleen. PMN were significantly higher at 3 dpi in SchuS4 infection. NK1.1^+^ cells were slightly higher in LVS infection, but reduced in SchuS4 infection (S2B Fig). CD3^+^T and B220^+^B cells were unchanged in lungs (S2B Fig) or spleen until 6 dpi. However, CD8^+^T cells in lungs increased at 9 dpi in LVS infection. Thus, consistent with elevated chemoattractants, Ft elicits an acute infiltration of, primarily, myeloid cells in lungs and spleen.

To better understand this acute inflammatory process, sequential tissue pathology was considered. Ft-infected lungs had typical lobar pneumonia including focal congestion by 3 dpi and unilateral or bilateral consolidation at 6 dpi (Fig 2B). Mediastinal lymph nodes were enlarged and palpable at 6 dpi. Spleens became enlarged, necrotic, and fragile in SchuS4 infection, but only enlarged in LVS infection. Numbers of spleen cells and spleen weight were both increased confirming splenomegaly (S2C Fig). Livers exhibited a few necrotic foci on the surface at 6 dpi. Histologically, SchuS4-infected lungs had solitary focal infiltrations by PMN at 1 dpi and many inflammatory foci consisting of PMN and mononuclear (MO) cells at 3 dpi. By 6 dpi, multiple inflammatory foci with mixed cellular infiltrates (PMN, MO/MΦ and lymphocytes), massive necrosis, and serous to fibrinous exudates or debris in alveoli/airways were present (Fig 2C). Inflammatory foci were mostly peri-vascular, peri-bronchiolar or in alveoli. Histological changes indicative of acute lung injury including PMN infiltration (of vascular, interstitial, and alveoli), diffuse alveolar damage, and hyaline membrane-like structures lining the alveoli were seen at 3 dpi (Figs 2C and S2E). Granulomas, marked thickening of inter-alveolar septa, and clusters of gram-negative bacteria also were seen (S2E Fig). In SchuS4-infected spleen, marginal zone thickening, red pulp inflammation, granulomas, and necrotic foci were prominent by 6 dpi (Fig 2C). Focal liver infiltration by PMN and MO was present at 3 dpi, with granuloma and necrosis at 6 dpi (S2F Fig). In LVS-infected lungs, PMN infiltration was low at 1 dpi, but multiple inflammatory foci containing PMN and MO were evident by 3 dpi with necrotizing inflammation at 6 dpi (Fig 2C). Necrotizing inflammation was prominent at 9 dpi. Marginal zone thickening and red pulp inflammation, without much necrotic changes, were seen in spleen (Fig 2C). Thus, the sequential pathology of SchuS4 and LVS infection are similar with development of mixed cellular inflammatory foci and ensuing necrotic lung damage. Notably, these pathological changes develop more rapidly in SchuS4 infection. Immunohistochemical staining also confirms the cellular infiltration by Ly6G^+^ PMN (S2G Fig, bottom panel) and Ly6C^+^ MO cells in Ft-infected lungs.

As the described inflammatory changes in the lung were consistent with acute lung injury, we further examined arterial blood oxygen saturation (SpO2) level in LVS-infected mice using pulse oximetry [40]. The level of SpO2 decreased in parallel with other indicators of lung inflammation with levels below approximately 75% preceding death (Fig 2D). This represents a significant loss of lung function, suggesting that respiratory failure is a key feature of mortality during Ft infection. BAL fluid total protein (Fig 2E) and cell number (S2D Fig) were significantly increased at 6 dpi, consistent with damage to the alveolar capillaries. These data demonstrate that acute inflammatory changes in the lung, accompanied by respiratory failure and damage to spleen and liver, likely results in the death of Ft-infected mice. In addition, during sub-lethal LVS (LD_50_, 500 cfu) infection where ~50% of infected mice survive (Fig 1C), pathological changes were less pronounced as reflected by lower tissue pathology scores (Fig 2F). Consistently, myeloid cell infiltration of the lungs was reduced in sub-lethal infection (Fig 2G). Further, intranasal instillation of 2 x 10^7^ iFt did not result in fatality or tissue pathology (S1I and S1J), suggesting that a threshold number of live Ft elicit a pathogenic host response. This observation also suggests that induction of a lethal acute inflammatory response requires sufficiently large numbers of live bacteria during Ft LVS infection. However, the innate inflammatory response is qualitatively and quantitatively different at below certain threshold numbers of LVS burden. Thus, overt innate inflammatory response induced by Ft likely result in necrotizing tissue damage and death during pulmonary tularemia.

### Medullary and extra-medullary myelopoiesis drives rapid and sustained cellular infiltration into tissues

**Fig 3:**
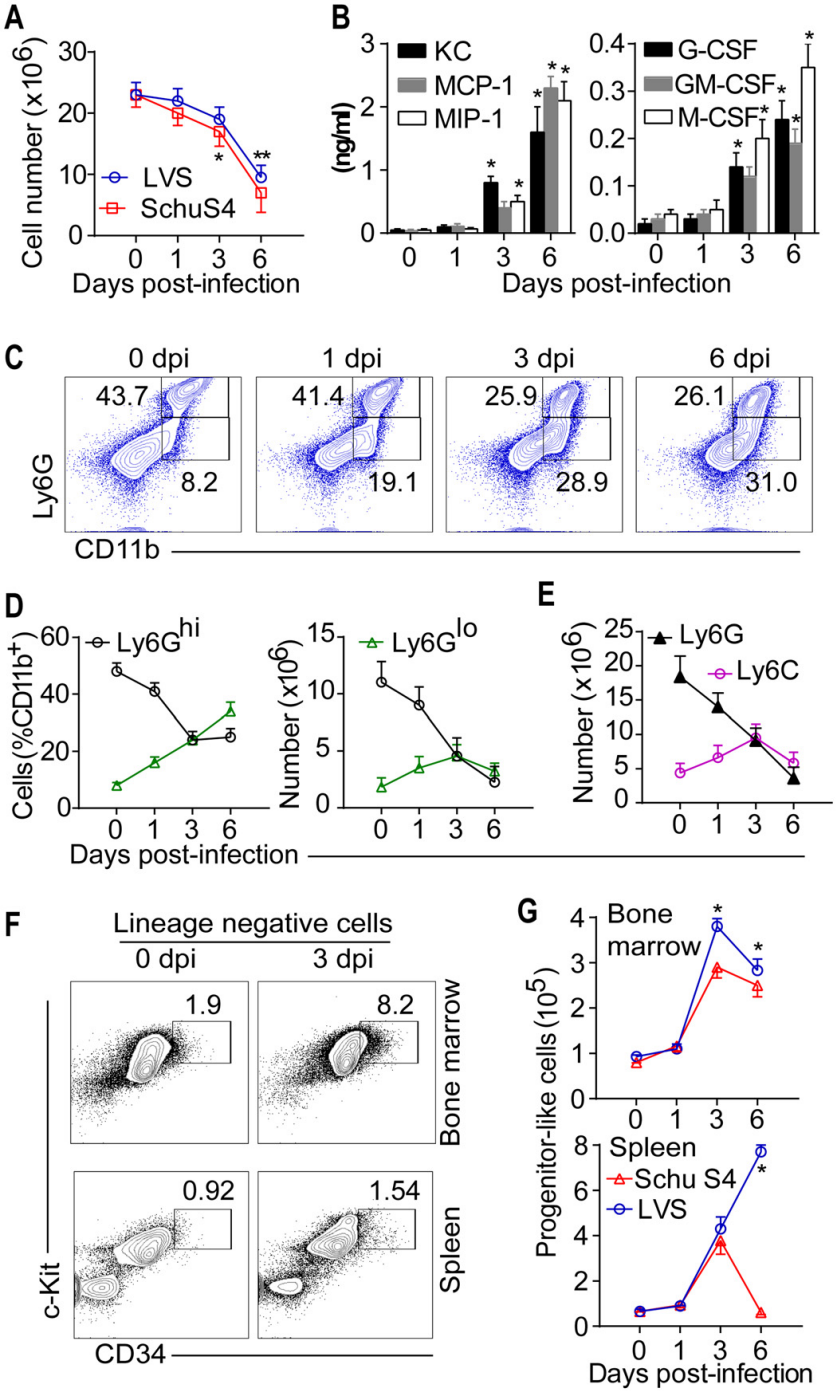
Medullary and extra-medullary myelopoiesis during lethal Ft infection. (A) Total number of BM cells in Ft infection (mean ± SD of two (SchuS4, n = 6 mice) or three (LVS, n = 9 mice) independent experiments, Student’s t-test, *p<0.05, **p<0.01). (B) Cytokine levels in BM aspirates of Ft LVS-infected mice (mean ± SD of two independent experiments, n = 6 mice, ANOVA test with Tukey’s post-test, *p<0.05). (C) Representative flow plots of bone marrow (BM) cells for analysis of myelopoiesis in Ft LVS infection. (D) Frequency/total numbers of Ly6G^hi^ and Ly6G^lo^ cells in BM and (E) total numbers of Ly6G^+^ and Ly6C^+^ cells in BM (mean± SD of three independent experiments, n = 9 mice, Student’s t-test, *p<0.05). (F) Representative flow plots for analysis of progenitor-like cells and (G) their numbers in BM and spleen during Ft infection (mean ± SD of two (SchuS4, n = 6 mice) or three (LVS, n = 9 mice) independent experiments, Student’s t-test, *p<0.05).

During systemic infection, the hematopoietic system responds by increasing myeloid cell production in the bone marrow (BM) and releasing myeloid cells into blood and tissues [41–43]. Thus, accumulation of myeloid cells in Ft-infected lungs suggests emergency myelopoiesis. Decreased total BM cells at 3 and 6 dpi (Fig 3A), the blanching appearance of the femur bones on gross examination, and the presence of larger myeloid precursors on histological sections of femurs (S3B and S3C Fig) further suggest an altered BM myelopoiesis. Consistent with increased emigration of myeloid cells from the BM compartment, the levels of KC, MCP-1, MIP-1, G-CSF, GM-CSF and M-CSF were significantly increased in BM aspirates (Fig 3B). Analysis of BM cells revealed a decrease in mature neutrophils (CD11b^+^Ly6G^hi^) at 1 and 3 dpi, while immature neutrophils (CD11b^+^Ly6G^int^) increased progressively (Fig 3C and 3D), consistent with observations of emergency myelopoiesis [41]. In addition, the frequency of monocytes (CD11b^+^Ly6C^+^) was increased at 3 dpi, but decreased at 6 dpi (Fig 3E). Moreover, flow cytometric analysis for myeloid progenitor cells [41, 43] revealed an increased frequency of Lin-Sca-1+CD34+c-kit+ progenitor-like cells in the BM at 3 and 6 dpi compared to controls (Figs 3F, 3G and S3A).

Extra-medullary hematopoiesis (EMH) is a conserved innate immune mechanism [43]. Splenomegaly combined with increased total spleen cells in pulmonary tularemia suggests EMH. Consistently, enlarged spleens revealed histological evidence of EMH, including the presence of megakaryocytes and increased myeloid precursors (S3D Fig). Increased numbers of Lin-Sca-1+CD34+c-kit+ progenitor-like cells were seen in LVS-infected spleen at 3 and 6 dpi (Figs 3F, 3G and S3A). However, while this was similar in spleens of SchuS4 infected mice, most progenitor cells were absent by day 6 post-infection (Fig 3G), potentially a consequence of massive splenic necrosis and splenocyte death. In contrast to spleens of LVS infected mice, most of the splenic structures were necrotic in SchuS4 infection by day 6 (Fig 2C), suggesting that splenic damage may be an important feature accelerating death during SchuS4 infection. Thus, during lethal pulmonary tularemia, medullary myelopoiesis and splenic EMH supports continued recruitment of myeloid cells, driving a sustained accumulation of myeloid cells in tissues.

### Ft elicts recruitment of immature myeloid cells (IMC) that exhibit an MDSC phenotype

**Fig 4:**
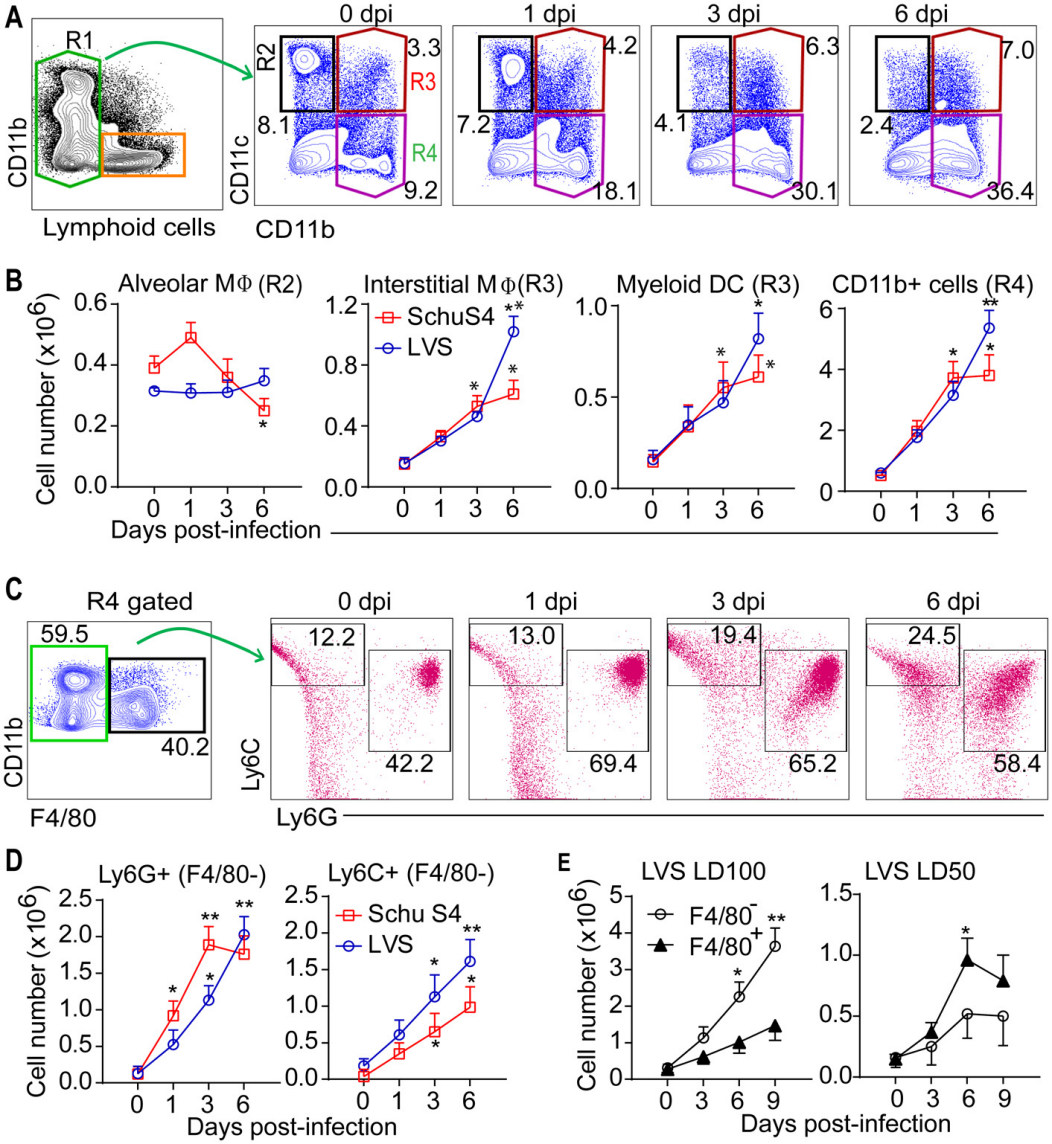
Ft elicts recruitment of IMC with MDSC phenotype. (A) Flow plots of multiple myeloid cell analysis scheme in in Ft LVS-infected lungs. Ft-infected lung cells were stained for cell-specific markers (see Methods); lymphoid cells were excluded and cells in R1 gate were analyzed for CD11b and CD11c expression. (B) Cell numbers of myeloid subsets from LVS or SchuS4 infected mice were compared with control mice (0 dpi) using gating strategy as shown in S4A–S4G Fig(mean ± SD of two (SchuS4, n = 6 mice) or three (LVS, n = 9 mice) independent experiments, Student’s t-test, *p<0.05 and **p<0.01 indicate differences at various dpi). (C) Cells from R4 gate were analyzed for F4/80, Ly6G and Ly6C expression. (D) Cell numbers of Ly6G^+^ and Ly6C^+^ cells from F4/80^-^ population in LVS or SchuS4 infected mice (mean ± SD of two (SchuS4, n = 6 mice) or three (LVS, n = 9 mice) independent experiments, Student’s t-test, *p<0.05 and **p<0.01 indicate differences at various dpi). (E) Cell numbers of F4/80^+^ and F4/80^-^ cells (R4 gated) in lungs following lethal (1000 cfu) or sub-lethal (500 cfu) LVS infection (mean ± SD of two experiments, Student’s t-test, *p<0.05 indicates a difference between LD_100_ and LD_50_).

Despite progressive recruitment of innate myeloid cells to lungs, Ft replication continues and necrotizing inflammation ensues. While supporting Ft replication, recruited myeloid cells are likely impairing protective responses and/or bacterial clearance. A detailed analysis was done to identify the multiple myeloid cell population that infiltrate lungs and spleen by using multi-parameter flow cytometry as described previously [44], with a slight modification (Figs 4A and S4A–S4G). In lethal pulmonary tularemia, alveolar MΦs (R2) were reduced somewhat with SchuS4 infection, while for both strains interstitial MΦ and myeloid DC (R3) increased (Figs 4B and S4H). Interestingly, infiltrating CD11b^hi^ (R4) cells increased progressively (Figs 4B and S4H). Among CD11b^hi^ cells, F4/80^hi^ inflammatory MΦ (R6) were reduced to ~20%, but F4/80^-^ immature cells (R5) increased from 60 to 80%. Among F4/80^-^ cells, putative PMN-MDSC (CD11b^hi^Ly6G^hi^Ly6C^int/low^) and MO-MDSC (CD11b^hi^Ly6C^hi^Ly6G^-^) were identified as described previously [45–46], and total numbers of these cells were increased significantly in lungs (Fig 4C and 4D), BAL fluid (S4I Fig) and spleen. In contrast, during sub-lethal infection, F4/80^hi^ cells increased, while F4/80^-^ cells decreased (Fig 4E), suggesting that survival of mice may result from effective recruitment and/or maturation of inflammatory MΦ, while IMC/MDSC predispose to tissue damage and death.

**Fig 5:**
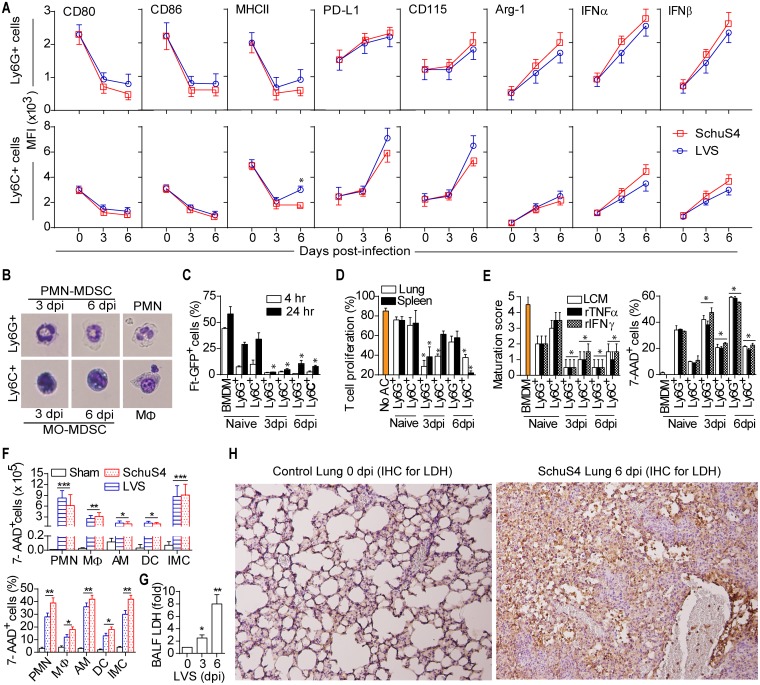
Ft-elicited myeloid cells are IMC/MDSC. (A) MFI of the indicated myeloid cell marker expressed in Ly6G^+^ or Ly6C^+^ cells in lungs during Ft infection (mean ± SD of two (SchuS4, n = 6 mice) or three (LVS, n = 9 mice) independent experiments; MFI values for all the markers in Ft-infected mice at 3 and 6 dpi were significantly (p<0.05) different from control mice at 0 dpi. *p<0.05 indicates difference between SchuS4 and LVS). (B) Giemsa staining of isolated Ly6G^+^ or Ly6C^+^ cells from LVS-infected lungs. Note immature nuclear morphologies of ring/band-shaped (Ly6G^+^) or small round (Ly6C^+^) nuclei. (C) Percent Ly6G^+^ or Ly6C^+^ cells positive for phagocytosed Ft-GFP upon *in vitro* infection. (D) *In vitro* proliferation of T cells (CFSE dilution) with and without accessory cells (AC) such as Ly6G^+^ and Ly6C^+^ cell populations. (E) *In vitro* differentiation/maturation score and 7-AAD positivity for Ly6G^+^ or Ly6C^+^ cells and frequency of 7-AAD^+^ Ly6G^+^ and Ly6C^+^ cells cultured with indicated factors (For C-E, mean ± SD of two independent experiments, Student’s t-test, *p<0.05). (F) Numbers/frequency of 7-AAD^+^ myeloid cell subsets from Ft-infected lungs at 6 dpi (mean ± SD of two (SchuS4) or three (LVS, Sham) independent experiments, Student’s t-test, *p<0.05, **p<0.01, ***p<0.001). (G) LDH activity in BAL fluid following LVS-infection (mean ± SD of two independent experiments, Student’s t-test, *p<0.05, **p<0.01). (H) Positive immunoreaction for localization of LDH, as an indicator of necrosis, in lung sections from control or SchuS4-infected mice (IHC with hematoxylin counterstaining, 400x).

Putative PMN-MDSC (Ly6G^hi^) and MO-MDSC (Ly6C^hi^) cells had reduced expression of CD80, CD86, and MHCII, but increased expression of PDL-1, CD115 (CSF-R), Arg-1, IFNα and IFNβ (Fig 5A), confirming an MDSC phenotype. In contrast, Ly6C^+^ and Ly6G^+^ cells from uninfected mice (day 0) expressed CD80, CD86 and MHCII, but lack PD-L1, CD115, and Arg-1. Moreover, nuclear morphology of PMN-MDC (ring-shaped/band) and MO-MDSC (small round) confirm that these cells are IMC and distinct from mature PMN (poly-segmented nuclei) and MΦ (large round nuclei) (Fig 5B). Consistently, many IMC were seen *in situ* in lung tissues at 6 dpi (Figs 2C and S2G) and in cytospin cell smears of BAL fluid at 6 dpi (S4I Fig). Thus, the infiltrating CD11b^hi^Ly6G^hi^ or Ly6C^hi^ cells are phenotypically IMC/MDSC.

Functionally, PMN- and MO-MDSC cells were less phagocytic than naïve BMDM or MΦ (Figs 5C and S5A). PMN-MDSC (Ly6G^+^) from lung/spleen at 3 dpi, but not those at 6 dpi, also inhibited *in vitro* T cell proliferation (Figs 5D and S5B) as did MO-MDSC (Ly6C^+^) from lungs (both 3 and 6 dpi) and spleen (6 dpi). In addition, very few isolated IMC harbor bacteria (6.2 ± 0.9 x 10^4^ Ft/million cells, n = 6 mice), which may reflect reduced *in vivo* phagocytic activity. Thus, these cells are *bona fide* MDSC. Moreover, *in vitro* maturation of isolated PMN-MDSC was impaired and many were dead (7-AAD^+^) at 48 h compared to control cells (Fig 5E). Although many MO-MDSC did differentiate, significant numbers also were dead (Figs 5E and S5C). These results suggest that PMN- and MO-MDSC remain immature and may die *in vivo*. Indeed, in Ft infected lungs, about 30–40% of PMN, IMC or AMΦ as well as 10–20% of MΦ or myeloid DC were dead at 6 dpi (Fig 5F). About 10% of these cells were also TUNEL^+^. The LDH activity in BAL fluid was increased (Fig 5G) and immunohistochemical localization for LDH in lung sections revealed positive immunoreaction at inflammatory foci (Fig 5H), confirming necrosis in the lungs. Greater or more rapid myeloid cell death may reflect the extensive tissue necrosis and earlier demise with SchuS4 infection. As such, massive necrosis with abundant dead cells was seen in the lungs and spleen of SchuS4 infected mice (Fig 2C). Thus, during Ft infection, large numbers of IMC/MDSC are recruited to the lung with limited capacity for phagocytosis or further maturation, but a propensity to die. Further, myeloid cell death in the lung is substantial and temporally associated with necrosis and loss of pulmonary function, suggesting the likelihood of a causal link between these processes.

### IMC/MDSC associate with tissue pathology and host mortality in pulmonary tularemia

**Fig 6:**
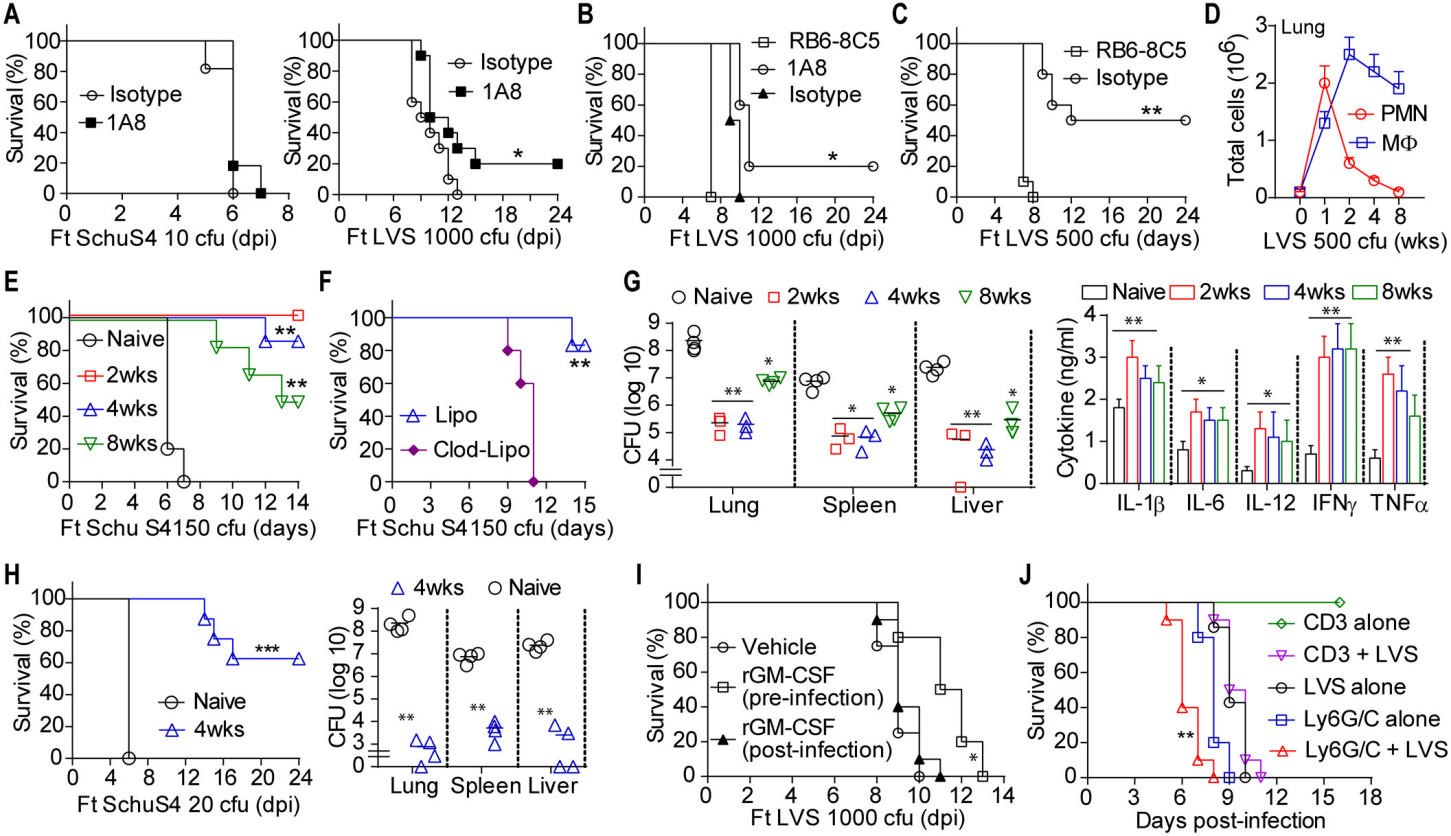
IMC/MDSC are associated with tissue damage and mortality during pulmonary tularemia. (A) Survival of LVS or SchuS4 infected mice depleted of myeloid cells. Ft-infected mice were treated with anti-Ly6G (clone 1A8) antibody or its isotype control antibody (200 μg/mouse/daily once) by intraperitoneal injection once daily between 2 to 4 dpi (% survival of two independent experiments, n = 10 mice, Log rank (Mantel-cox) test, *p<0.05). (B) Survival of LVS (1000 cfu/ lethal dose) infected mice depleted of myeloid cells. Mice were treated with RB6-8C5 antibody, 1A8 antibody or isotype control antibody (200 μg/mouse/daily once) by intraperitoneal injection at -1 (a day prior to infection), 2 and 4 dpi (% survival of two independent experiments, n = 10 mice, Log rank test, *p<0.05). (C) Survival of LVS (500 cfu/sub-lethal dose) infected mice depleted of myeloid cells. Mice were treated with RB6-8C5 antibody or isotype control antibody (200 μg/mouse/daily once) by intraperitoneal injection at -1 (a day prior to infection), 2 and 4 dpi (% survival of two independent experiments, n = 10 mice, Log rank test, **p<0.01). (D) Numbers of matured myeloid cells (PMN and macrophages) in lungs of sub-lethal LVS-infected survivor mice (mean ± SD of two independent experiments, Student’s t-test, *p<0.05, **p<0.01). (E) Survival of mice challenged with SchuS4 (150 cfu). Mice were first infected with sub-lethal LVS infection and those surviving mice were challenged with SchuS4 at 2, 4 or 8 weeks post-LVS infection and monitored for survival (% survival of two experiments, n = 6–10 mice, Log rank test, *p<0.05, **p<0.01). (F) Survival of mice challenged with SchuS4 (150 cfu). Mice were first infected with sub-lethal LVS infection and those surviving mice were treated with either clodronate-liposome or liposome vehicle alone at the end of 3^rd^ week (2 days apart) and re-challenged with SchuS4 at the end of 4^th^ weeks post-LVS infection for monitoring the survival (% survival, n = 6 mice/group, Log rank test, ***p<0.001). (G) Tissue bacterial burden and lung cytokines at 15 days post-SchuS4 challenge in surviving mice from experiments as shown in Panel E mean ± SD of two experiments, n = 4–6 mice, Student’s t-test, *p<0.05, **p<0.01). (H) Mice surviving sub-lethal LVS infection were challenged with 20 cfu of SchuS4 at 4 weeks post-LVS infection and monitored for survival and tissue bacterial burden was estimated at 36 days post-SchuS4 challenge (% survival or mean ± SD of 8 mice, Log rank test, **p<0.01, ***p<0.001). (I) Survival of LVS-infected mice treated with rGM-CSF. Either mice were treated intranasally with rGM-CSF (1 μg/mouse/daily once) pre-infection at -7, -5, -3, and -1 dpi or treated with rGM-CSF (2 μg/mouse/daily once) post-infection by intraperitoneal injection at 2 and 4 dpi (% survival of two independent experiments, n = 10 mice, Log rank test, *p<0.05 indicates the mean time-to-death between GM-CSF treated and untreated mice). (J) Adoptive transfer of Ly6G/C^+^ cells (isolated from lungs of LVS-infected mice at 6 dpi) or CD3+ T cells (isolated from spleen of naïve mice) into donor mice and monitored for survival following with and without subsequent LVS infection. The Ly6G and Ly6C fractions of myeloid cells were isolated from the lungs of LVS-infected donor mice (CD45.1) at 6 dpi (see methods) and equal numbers of these cells (2 x10^6^ in 2 ml PBS) were mixed together and treated with antibiotic ciprofloxacin (100 μg/ml) for 45 min to kill cell-associated bacteria. After washing twice with PBS, cells were re-suspended in 50μl PBS and were transferred to naïve recipient mice (CD45.2) by intratracheal intubation. The CD3+ T cells were isolated from spleen of naïve mice and transferred to recipient mice as above. These mice were either infected with LVS on following day or left uninfected for monitoring the survival (% survival of two independent experiments, n = 10 mice, Log rank test, **p<0.01 indicates the difference between Ly6G/C + LVS group *vs* LVS alone group).

Neutrophils are critical for protection against intradermal Ft infection [12], but acute infiltration and death of IMC/MDSC in lungs might be detrimental in pulmonary tularemia. To test this, we depleted Ly6G^+^ cells (immature and mature neutrophils) using anti-Ly6G antibody (clone 1A8) [47,48]. Gr-1^+^ cells were greatly reduced in lungs, spleen, and blood at 4 and 6 dpi (S6A Fig). Although most Ly6G^+^ cells were depleted, a number of Gr-1^low^ cells remain in lungs and blood, consistent with maintenance of monocytic Ly6C^+^ cells [47]. Ly6G-depleted mice succumbed to SchuS4 infection with typical kinetics, while 20% survived LVS infection (Fig 6A). The anti-Ly6G (1A8) antibody does not target Ly6C^+^ cells that lack Ly6G such as immature and mature monocytes [47,48]. To consider the contribution of all myeloid cells, we also depleted Gr-1+ cells (Ly6G and Ly6C) using RB6-8C5 ab. The RB6-8C5 antibody reduces MDSC expansion and can enhance immunity during microbial infections [49, 50]. Ly6G+ cells were almost completely depleted and Ly6C+ cell numbers were significantly reduced in the RB6-8C5 antibody-treated group (S6B Fig). In contrast to 1A8 antibody-treated mice, RB6-8C5 antibody-treated mice succumbed more rapidly to lethal LVS infection (with a mean time to death of 7 days) versus mice receiving isotype control antibody (Fig 6B), suggesting that some Gr-1^+^ cells contribute to the limited resistance of these mice to lethal Ft LVS infection. In 1A8 antibody-treated mice, Gr-1^hi^ cells (Ly6G^+^ cells) are depleted, suggesting that while immature and mature neutrophils are depleted, remaining Gr-1^lo^ monocytes likely contribute to protection. Similarly, RB6-8C5 antibody-treated mice infected sub-lethally with Ft LVS (Fig 6C) were uniformly susceptible, dying by 8 dpi. The observation that RB6-8C5-treated mice lethally or sub-lethally infected with Ft die more rapidly than isotype control antibody-treated mice suggests that a Gr-1^+^ population of cells provides some defense against Ft. Consistent with this, blocking the recruitment of mature Ly6G^hi^ (mature) neutrophils to the lung in LVS infected mice via systemic G-CSF neutralization increased both the proportion and number of Ly6G^low^ (immature) neutrophils, but shortened their mean time to death (S6C and S6D Fig). Collectively, these data suggest that mature neutrophils and/or macrophages are likely essential for clearance of Ft, while overwhelming numbers of immature myeloid cells (IMC) contribute to mortality.

If recruitment of dying IMC to lungs is a primary factor in pulmonary tularemia mortality, eliciting mature myeloid cells should be protective. About 50% of mice survive past 14 dpi following sub-lethal infection (Fig 1C) and lung infiltrating IMC are markedly reduced (Figs 2G and 4E). In contrast to lethal pulmonary tularemia where immature PMN predominate over immature MΦ (5:1), mature PMN predominate over mature MΦ (3:1) at 3 and 6 dpi in sub-lethal infection (Fig 6D). Although the frequency of AMΦ is initially reduced, it was increased in sub-lethal infection by 9 dpi, but not in lethal infection. Further, the extent and severity of inflammation and tissue necrosis are significantly reduced (Fig 2F), while bacterial burden at 3 dpi is lower, but is equivalent at day 6 (S6E Fig). Among mice surviving sub-lethal infection, mature MΦ predominated over PMN (3:1) in lungs at 4 or 8 weeks (Fig 6D), while numbers of T and B cells were slightly increased (S6F Fig), but no viable bacteria or tissue pathology was present after 2 weeks p.i (S6G Fig). Finally, as noted above, Gr-1^+^ cells are required for this protection as myeloid cell depletion with anti-Gr-1 abrogates survival (Fig 6C). Thus, the absence of sustained IMC recruitment correlates with host survival in sub-lethal infection, while mature cells appear protective, further suggesting that the IMC/MDSC response plays a key role in acute pathology and death in lethal tularemia.

If IMC recruitment is pathogenic, the mature myeloid cell response elicited during sub-lethal LVS infection might protect mice from lethal SchuS4 challenge. To test this, survivors were re-challenged with LVS (10^4^ cfu) or SchuS4 (150 cfu) at 2, 4, or 8 weeks post-infection. As expected, there was no mortality observed upon LVS re-challenge, consistent with development of adaptive immunity to LVS. Notably, all SchuS4 re-challenged mice survived beyond 8 dpi (Fig 6E). Mice challenged at 2 weeks were protected through 14 dpi, while those challenged at 4 or 8 weeks exhibited 20% and 50% mortality, respectively. As this protection is transient, it is unlikely to result from durable antibody responses and is more likely due to transient mature myeloid cell responses. Further, clodronate treatment of survivors to deplete phagocytes rendered them susceptible to SchuS4 infection (Fig 6F), suggesting that these cells are necessary for protection. Bacterial burden was significantly reduced in re-challenged mice at 15 dpi in comparison to naive mice infected with SchuS4 alone at 6 dpi (Fig 6G). Notably, lung TNFα, IFNγ, IL-1β, IL-6, and IL-12p40 was substantially increased in re-challenged mice (Fig 6G), consistent with the presence of responsive and mature MΦ. Moreover, about 60% of mice re-challenged at 4 weeks with 20 cfu SchuS4 survived beyond 24 dpi and bacteria were cleared at 36 dpi (Fig 6H). Thus, mature myeloid cells appear sufficient to control lethal Ft infection and also account for survival of mice during sub-lethal infection.

Recombinant GM-CSF can reverse monocyte deactivation in sepsis and rescues patients from sepsis-associated immunosuppression [51] and rGM-CSF administration facilitates alveolar macrophage-dependent protection in mice infected with influenza virus [52]. To evaluate whether GM-CSF might promote a protective response to Ft, we treated mice with rGM-CSF either pre-infection (at -7, -5, -3 and -1 days as to elicit myeloid cell responses before infection) or on days 2 and 4 post-infection to enhance the myeloid cell response. While neither regimen protected mice from lethal LVS infection, the mean time-to-death was significantly longer in pre-infection treatment group (Fig 6I). Consistent with our results suggesting a protective role for mature myeloid cells, significantly more alveolar macrophages and neutrophils were present in pre-infection treated mice on the day of infection (S6H Fig). However, in the post-infection treatment group, rGM-CSF did not change the number or type of cells recruited to the lung at 6 dpi (S6H Fig). Neither group exhibited improved bacterial clearance in lungs or spleen at 6 dpi (S6I Fig). These results suggest that while resident, mature myeloid cells have some protective capacity, once initiated, the IMC/MDSC response to Ft cannot be overcome by rGM-CSF. Further, the prolonged survival of pre-infection rGM-CSF-treated mice did not correlate with reduced bacterial burden at 6 dpi, suggesting that host death is not strictly correlated with bacterial load and supporting the concept that a certain threshold of bacterial numbers is needed to elicit a lethal IMC host response.

Finally, adoptive transfer of Ly6G^+^ and Ly6C^+^ IMC/MDSC fractions from LVS-infected mice (6 dpi) exacerbated mortality in naïve mice following LVS infection with a significantly shorter mean time-to death (Fig 6J) and extensive lung pathology (S6J and S6K Fig). In addition, transfer of these cells without subsequent infection also caused death in naïve recipient mice, with significant lung pathology (Figs 6J and S6J and S6K). However, adoptive transfer of similar numbers of naïve CD3+ T cells did not result in mortality. Collectively, these data indicate that acute pathology and host mortality in pulmonary tularemia result from a sustained recruitment of IMC/MDSC.

## Discussion

Subversion of host recognition and antimicrobial processes by Ft which may alter innate and adaptive immunity has received considerable attention (reviewed in [2]). Ft uses various stealth mechanisms to exploit host cells and facilitate its replication, leading to the perspective that unfettered bacterial growth kills the host. Our results demonstrate that Ft burden alone is insufficient to kill mice and that excessive inflammatory cytokines are an unlikely explanation for death. Instead, our data reveal that an overt host cell inflammatory response consisting of predominantly IMC/MDSC which die in the lung and likely important for temporally associated necrotic lung damage, loss of lung function, and acute death of the host.

Time to death in pulmonary tularemia is not dose-dependent for the virulent SchuS4 strain and requires a ‘window’ of 6 days. This 6 day period is also evident for the LVS strain after infection with 10^5^−10^7^ bacteria. During this time the host cellular inflammatory response leads to accumulation and death of IMC resulting in tissue damage and host death. Although Ft evokes this overwhelming innate response, a particular threshold of live bacteria appears to be required, as sub-lethal numbers of bacteria do not induce an influx of IMC. Instead, sub-lethal infection elicits mature phagocytes capable of clearing the infection and promoting survival. Thus, the bacterial cfu, bacterial strain, and/or mouse strain may influence whether the cellular response is protective or detrimental. Similarly, attenuation but not complete ablation of the neutrophil-driven inflammatory response protects mice during sub-lethal compared to lethal flu infection [53, 54].

Initial delay in inflammatory cytokines, but early appearance of regulatory cytokines provoked the idea that Ft establishes an anti-inflammatory milieu for intracellular replication and survival [3, 6, 16–18]. This thought is somewhat paradoxical as early infiltration of neutrophils in Ft-infected tissues was described over 40 years ago [9, 10] and neutrophils have been considered protective [12, 13]. Intriguingly, large numbers of neutrophils are recruited during Ft infection, but Ft itself limits neutrophil oxidative burst and other microbicidal machineries [2] thereby facilitating evasion of traditional host defenses known to restrict bacteria. Our data suggests that while accumulating neutrophils might play a role in controlling bacterial growth, they also likely contribute to host tissue damage. We previously demonstrated that overabundant lung infiltrating neutrophils are detrimental in tularemia as *Mmp9* ^-/-^ mice exhibited diminished leukocyte recruitment, reduced bacterial burden, and increased survival versus Wt mice [11]. Previous literature ‘painted’ a conflicting picture as to whether neutrophils are protective or detrimental during tularemia. In light of our findings, the phenotype and number of neutrophils that accumulate may determine which role predominates. Indeed, Gr-1+ cells are necessary for protection, but only under sub-lethal conditions when mature myeloid cells predominate. However, and more importantly, our data are the first to reveal that the cellular inflammatory response in the lung, including recruitment of numerous IMC/MDSC, plays a detrimental role in mediating tularemia pathogenesis. The presence of immature neutrophils provides some explanation for why neutrophils are abundant but ineffective at clearing Ft. The accumulation of immature neutrophils also might reflect elevated levels of MMP-9 (which facilitates neutrophil recruitment) or reduced α1-antitrypsin (which checks neutrophil elastase activity) in lethal pulmonary tularemia [11, 55]. Curiously, high MMP-9 activity, low α1-antitrypsin production, and unrestrained serine protease (elastase) activity are features of the promyelocyte and metamyelocyte/band stages of neutrophil development that may contribute to irreversible necrotic tissue damage [56]. Further, recruitment of immature neutrophils may explain why elevated IL-17 in the absence of IL-10 leads to neutrophil accumulation and lung pathology with no change in control of bacterial numbers, but IL-10^-/-^ mice depleted of neutrophils with the Ly6G antibody (clone 1A8) survives longer [57]. While neutrophil granular proteases may account for organ damage, other protease-independent consequences of cell death (e.g. DAMPs and alarmins) might also signal epithelial cell death. Nevertheless, in Ft infected mice 30–40% of lung neutrophils (mature and immature) are dead by 6 dpi, providing significant opportunity for epithelial damage. This time frame correlates with and may account for the observed 6 day mortality “window” in infected mice.

Later in pulmonary tularemia, the release of inflammatory cytokines (IL-1β, IL-6, TNFα, etc.) and DAMPs/alarmins (e.g. HMGB1, S100A9) are suggested ‘drivers’ of host death [4, 5]. However, other studies and our results suggest protective roles for these cytokines in tularemia [37, 38]. In addition, during pulmonary tularemia, HMGB1 neutralization prolonged time to death, but did not protect mice [58]. Instead, tissue damage due to overt IMC recruitment appears to account for host mortality. In contrast, eliciting a mature myeloid cell response in lungs by prior-LVS (LD_50_) infection confers dramatic, transient, and likely innate protection against SchuS4. Of note, the mature myeloid cell response to sub-lethal LVS infection appears similar in all mice observed, yet half of these mice still die. It is unclear whether death under these conditions is due to dying IMC present at some significant number, but with lower frequency or other distinct mechanism. Antibodies generated during prior infection with LVS could account for significant protection during re-challenge with SchuS4. However, LVS-specific serum antibodies are not effective against SchuS4 infection in mice [2, 59]. Nevertheless, a transient cytokine and myeloid cell response elicited by poly(I:C) significantly prolonged mice survival in pulmonary tularemia [60], which is consistent with our findings. In addition, the IMC/MDSC response may be elicited independently from (or interfere with) adaptive responses and may help explain why Ft vaccination is not long-lasting or, otherwise, unsuccessful. Thus, eliciting appropriate mature myeloid cells can effectively clear Ft infection, while accumulating IMC are detrimental in pulmonary tularemia.

**Fig 7:**
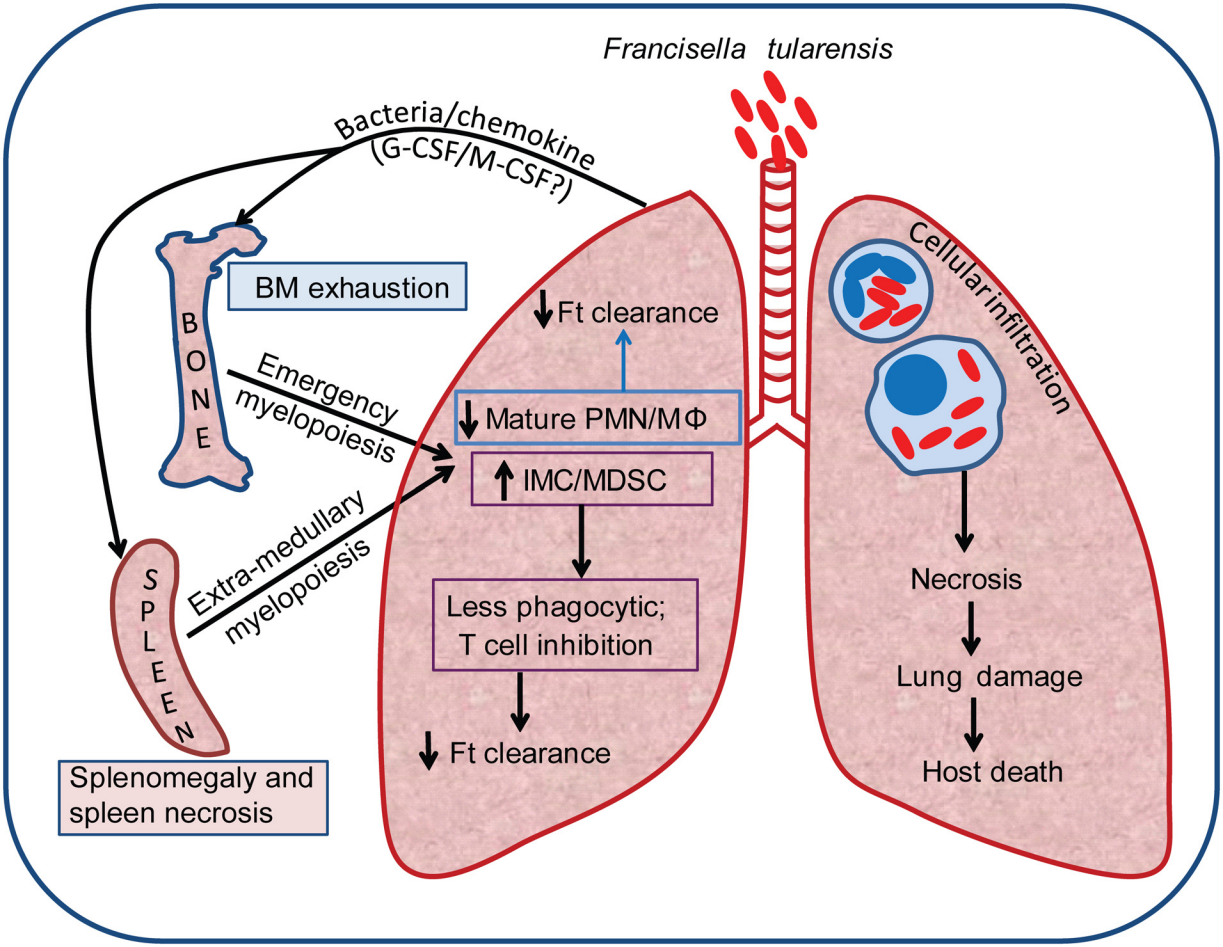
A schematic diagram shows the model for pathogenesis of lethal pulmonary tularemia in mice. Ft elicits an overt inflammatory change by recruitment of a large proportion of IMC/MDSC to lungs and spleen, where these cells die and likely drive irreversible necrotic tissue damage and multi-organ dysfunction leading to host death. Later, Ft-elicited myelopoietic response appears to exhaust production of myeloid cells by the BM and spleen leading to failure of immune response to control Ft replication.

In lethal pulmonary tularemia, a majority of the infiltrating myeloid cells are IMC/MDSC capable of inhibiting T cell proliferation. MO-MDSC inhibited T cell proliferation at higher number than PMN-MDSC suggesting differences in longevity or cell-type specific functions of MDSC [61]. While recruited MDSC die in the lung, their impact on specific T cell function during lethal Ft infection is unknown. However, it is intriguing that MDSC develop within a few days of pulmonary infection. This may not be surprising as high frequencies of MDSC have been reported in acute bacterial infection/sepsis models and chronic microbial infections [20–30]. In these conditions, MyD88/NF-kB signaling-mediated G-CSF is essential for progenitor expansion and PMN-MDSC skewing, while IL-1, IL-6, TNFα or S100 are required for activation of MDSC in target organs, with these cells inhibiting Th1 and CD8+ T cells and exhibiting defective phagocytosis but undergoing accelerated apoptosis [23–31]. These studies suggest a universally conserved mechanism for BM depletion; emergency myelopoiesis, IMC expansion and MDSC activation in peripheral organs, during microbial infections [41, 42]. A similar mechanism appears to be activated with Ft infection resulting in accumulation of IMC/MDSC in lungs and spleen. More specifically, early activation of chemokines and myeloid cell growth factors likely drive the initial recruitment of neutrophils and some monocytes by 2–3 dpi. This is followed by activated BM myelopoiesis and release of an apparent majority of IMC into lungs by 3 dpi onwards with depletion of BM cells and compensatory activation of extra-medullary myelopoiesis in spleen by 6 dpi [62]. These cells are poor phagocytes and die in the lung, a catastrophic event temporally associated with lung damage, loss of lung function, and death of the host (Fig 7). While our results are very similar to what has been described by Moldawer and other groups in regard to IMC/MDSC expansion, rGM-CSF administration at post-infection was not beneficial, suggesting that reactivating monocytes or relieving other immunosuppressive features may not occur or might be insufficient to overcome the developing pathology in pulmonary tularemia.

Despite conferring a survival advantage in LVS infection, depletion of Ly6G^+^ cells with the 1A8 antibody had no impact on survival of SchuS4 infected mice. However, in these experiments the possibilities of simultaneously depleting mature neutrophils (which might facilitate bacterial clearance) and antibody-mediated cell destruction (which could contribute to host mortality) cannot be readily excluded. Thus, it is unclear if these results reflect a meaningful difference between Ft strains, limited usefulness of antibody-depletion to ascertain the role of myeloid cells, or unknown complexities of the role played by Ly6G^+^ cells. The increased susceptibility to Ft LVS seen with RB6-8C5 antibody treatment likely reflects the depletion of mature neutrophils and inflammatory monocytes which are insufficient in number to overcome a lethal infection, but required for survival during sub-lethal infection. Further investigation of the cells involved in protection and pathogenesis of pulmonary tularemia is needed. However, in contrast to the anticipated beneficial role of myelopoiesis in supporting host resistance [42, 62], recruited myeloid cells in pulmonary tularemia are dying and ensuing necrotizing inflammation associated with necrotic tissue damage. Although some studies suggest Ft inhibits apoptosis in human neutrophils, other studies report that Ft LVS and *F*. *novicida* induced apoptosis and pyroptosis in macrophages, and it remains possible that other forms of cell death (e.g., necrosis) could also be involved in pulmonary tularemia [63–68].

While we have demonstrated a mechanism of pathogenesis likely shared between LVS and SchuS4, our data does not directly explain why SchuS4 infected mice die more rapidly. Consideration of the possibilities may shed additional light on this important question. First, although bacterial burdens during SchuS4 infection are higher than those of LVS at 3 and 6 days post-infection (owing to the faster replication of SchuS4), escalating doses of SchuS4 do not further accelerate mortality. However, bacterial burdens in excess of those seen with SchuS4 can be achieved using 10^7^ LVS resulting in a time to death similar to that seen with SchuS4. Thus, the more rapid mortality seen with SchuS4 is almost certainly related to the faster replication of SchuS4, but the necessary window of at least 6 days demonstrates that a time-limited host-dependent process is also involved. Secondly, SchuS4 and LVS trigger very similar cytokine and chemokine kinetics with few exceptions, for example, lower MCP-1 and higher G-CSF in the lung as well as lower IFNγ and higher IL-6, G-CSF, and MCP-1 in serum. IL-6 and IFNγ are considered protective, so elevated serum IL-6 responses seem to be a poor explanation. Reduced production of IFNγ along with increased production of G-CSF (which during Ft infection appears to support immature neutrophil production) could be responsible, but relatively small differences in their concentration (an order of 200 pg/ml) would need to be critical. Finally, our data suggests that at a certain threshold of live bacteria, Ft elicits a myelopoietic response that appears to exhaust production of these cells by the BM and spleen leading to accumulation of dying immature myeloid cells in the lung. Although the above factors merit additional examination, this inappropriate innate response suggests a relatively straightforward possibility—the response elicited by LVS and SchuS4 are comparable—but by replicating faster, SchuS4 reaches the critical threshold at the soonest possible moment, initiating the 6 day long response that will result in death. This thinking suggests that the splenic failure seen with SchuS4 at 6 dpi, might occur with similar timing using larger quantities of LVS. Nevertheless, additional work will be required to better understand this and other mechanisms critical for the pathogenesis of pulmonary tularemia.

In summary, in lethal pulmonary tularemia, Ft elicits recruitment of a large proportion of IMC/MDSC to lungs and spleen, where these cells die and likely drive irreversible necrotic tissue damage, multi-organ dysfunction, and host death. The host response to sub-lethal infection is markedly different with greater recruitment and development of mature myeloid cells that are more effective at controlling Ft. This study defines critical parameters accompanying host death during pulmonary tularemia, identifies the inappropriate elaboration of IMC/MDSC that leads to necrotizing inflammation in multiple organs, as the Ft-driven cause of death, and suggests that differences in host mortality versus survival may be reflected, not primarily by control of bacterial load, but by the nature of the innate myeloid cell response during acute phase of infection.

## Materials and Methods

### Mice and bacteria

C57BL/6J wild-type, CD45.1 (B6.SJL-Ptprc^a^ Pepc^b^/BoyJ) congenic mice, and *Casp1/11* ^-/-^ (Casp1^tm1Flv^) (Jackson Laboratories) were housed in the Animal Resources Facility at Albany Medical College. Experiments were conducted using male and female mice (8–10 weeks). Ft SchuS4 and LVS were cultured in modified Muller Hinton (MH) or Brain Heart Infusion (BHI) broth as described [6]. All experiments utilizing SchuS4 were conducted within the Albany Medical College, CDC-certified BSL-3 facility.

### Ethics statement

All animals were maintained in the AAALAC accredited animal resource facility at Albany Medical College and handled in strict accordance with good animal practice as defined by the relevant international, national, and state animal welfare bodies. All animal work was approved by the Albany Medical College Institutional Animal Care and Use Committee (IACUC) in accordance with the Public Health Service Policy on Humane Care and Use of Laboratory Animals (ACUP #12–04001, 12–04002 and 12–04003).

### Intranasal Ft infection

Bacterial inocula were prepared by serial dilution in sterile PBS to defined cfu numbers. Mice were anesthetized with Ketamine (20mg/ml)/Xylazine (1mg/ml) mixture (80–100μl/mouse) and 40 μl of inoculum instilled in a single nare. An equal volume of inoculum was plated on MH chocolate agar to confirm cfu numbers. Sham-inoculated controls received an equal volume of PBS or appropriate vehicle medium.

### Oxygen saturation measurement

Arterial blood oxygen saturation (SpO2) was determined in uninfected and Ft-infected mice over the course of infection using a handheld pulse-oximeter (Edan Instruments USA, San Diego, CA). Mice were restrained by hand, covered with light blocking fabric supplied by the manufacturer, and held until calm (several seconds). The pulse-oximeter sensor placed on the hind leg paw or on the hind leg over the skin near the femur. Two to three minute readings were taken from each mouse and only readings that had no error codes were recorded. Control and uninfected (day 0) mice had mean SpO2 values of 100% +/- 0) by this method.

### Necropsy, tissue collection and histology/immunihostochemistry

Blood was collected by submandibular/facial vein bleeding as described previously [69], and mice were euthanized with Ketamine (20mg/ml)/Xylazine (1mg/ml) mixture (200μl/mouse) followed by cervical dislocation. Necropsy was performed, gross lesions were noted, and organs (lungs, liver and spleen) were collected aseptically to prepare tissue homogenate (for bacterial counting and/or cytokine measurements), single cell suspensions (for immunophenotyping), or histology (for pathological assessment) as described previously [6]. For lung homogenate preparation, either whole lungs or a half of the lungs containing pieces (consistent size for each mouse) from middle lobe, post-caval lobe, the right superior lobe and the left lung lobes were collected in sterile PBS. For histology, either the entire lung lobes or representative pieces each from the right superior and inferior lobes and a half of the left lung lobes were collected in 10% buffered formalin. Sections of entire lung lobes were examined for the location of inflammatory foci, type of infiltrating cells and the extent of necrotic changes in parallel with sections from uninfected or Ft-infected lungs and scored using the criteria detailed in Supplementary procedure (S1 Procedures). Either all or half of the spleen was collected in formalin for histology. Additionally, pieces of liver from left lateral lobe and medial lobe were collected in formalin. Tissues were processed by standard histological procedures and 4μm-thick sections were cut and stained with hematoxylin and eosin (HE). Immunohistochemical (IHC) analysis for cellular identity in lungs/spleen was performed using formalin-fixed paraffin-embedded tissue sections. Briefly, after de-parafinization, antigen retrieval was done with citrate buffer and the sample incubated with biotinylated antibodies (Biolegend) for Ly6G, Ly6C, or CD11b at 4°C for overnight. The next day, sections were stained using the ABC kit (Vectastain Elite, Vector Lab) and color developed with diaminobenzidin (DAB enhanced liquid substrate system, Sigma). For analysis of the extent of tissue necrosis, IHC for LDH localization was done using anti-LDH mAb (Abcam) antibody overnight and secondary-HRP conjugated antibody followed by DAB substrate (colorimetric), as above. Sections were counterstained with Hematoxylin-7211 (Richard-Allen Scientific).

### Bronchoalveolar lavage (BAL) fluid collection

BAL fluid was collected from control and Ft-infected mice. Briefly, mice were euthanized by ketamine/xylazine injection and cervical dislocation. Mice were immediately weighed to the nearest 0.1g. They were then exsanguinated by cutting the abdominal artery. Mouse lungs were then lavaged with 1 ml of sterile PBS containing proteinase-inhibitor and EDTA (0.05M) by gentle perfusion using hypodermic needle and syringe for 2–3 times. The lavage fluid (usually 400–700 μl) was recovered and transferred to microcentrifuge tubes on ice, spun at 1200rpm for 6 min, and the cell-free clear supernatant collected and used immediately for LDH assays or stored frozen at -80C for protein estimation. Cell pellets were re-suspended in the original volume of PBS and used for flow cytometry or cytospin smear preparation for differential cell counting (Giemsa).

### Bacterial burden

Either whole lungs (1 ml) or pieces of half lungs from different lobes (0.5ml) were placed in sterile tube containing PBS plus protease inhibitor cocktail (Roche Diagnostics, Indianapolis, IN) and were homogenized using sterile inert Zirconia beads in Mini Bead Beater (Biospec Products, Bartlesville, OK) for 3 cycle of 45 second with 1 min interval. Similarly whole spleen (1ml) or half spleen (0.5 ml) was homogenized. For the liver, 1/5^th^ of the liver from caudal and median lobes was homogenized in 1 ml of sterile PBS. After bead beating, the tissue homogenates were spin at 1500rpm for 3 min to settle the tissue debris. From the top clear homogenates, 50 or 100 μl was aliquoted for 10-fold serial dilution and at least 3 dilutions were plated onto MH chocolate agar. After 2 days, colony counts were performed and bacterial numbers (cfu) were calculated. Results are expressed as log_10_ cfu/ml/organ

### Cytokine/chemokine/eicanosoids measurement

Tissue homogenates, after aliquoting for bacterial burden assay, were spin at 12000rpm for 10 min to prepare debris/particulate-free clear homogenates. Cleared homogenates were stored frozen or processed immediately for cytokine/chemokine measurement. Lung homogenates were analyzed for cytokines and chemokines by using Mouse Group I and II Luminex assay kits (BioRad). In addition, measurement of IL-17A, TGFβ, IFNα (eBioscience), IFNβ (PBL Assay), PGE_2_ and LTB_4_ (Caymen) were performed using commercial ELISA kits.

### Multi-parameter flow cytometry for immunophenotyping

Myeloid cells types were evaluated by multi-color flow cytometry using a modification of the procedure described by Misharin et al. [43]. For preliminary immunophenotyping, single cell suspensions from collagenase-digested lung or spleen were surface stained with either lymphoid markers (CD3, CD4, CD8) or myeloid markers (CD11b, CD11c, F4/80, Gr-1) for 30 min. The cells were fixed in 1% paraformaldehyde prior to analysis. For detailed immunophenotyping to distinguish various myeloid cell types, the single cell suspensions of lungs or spleen were surface stained with biotinylated antibodies targeting lymphoid markers (CD3, CD4, CD8, NK1.1, B220, CD19, Terr119, and anti-biotin-fluorochrome as a ‘dump’ gate), myeloid markers (CD11b, CD11c, F4/80, Gr-1, Ly6C, Ly6G) and/or additional markers (CD80, CD86, MHCII, PD-L1 or CD115) for 30 min. Cells were fixed in 1% paraformaldehyde prior to analysis. For intracellular staining, fixed cells were permeabilized and stained with marker-specific or isotype control antibody for 30 min. Multi-parameter flow cytometry was performed on an LSRII (Becton Dickinson) and data analyzed using FlowJo software (v10.0.1) [6]. For myeloid cells analysis, lymphoid cells were first excluded from total lung cells and those remaining (R1) were gated into CD11c^hi^ (R2), CD11b^+^CD11c^+^ (R3) and CD11b^hi^ (R4) populations (S4 Fig). CD11c^hi^ cells (R2 region) were identified as alveolar MΦ (CD11c^+^CD11b^-^ F4/80^+^). Within the CD11b^+^CD11c^+^ population (R3 region), interstitial MΦ (F4/80^+^ Ly6C^+^MHCII^+^ Ly6G^-^) and myeloid DC (F4/80^low/-^ Ly6C^low/-^ MHCII^+^Ly6G^-^) and PMN-DC hybrid cells (F4/80^-^ Ly6C^-^ MHCII^low/-^Ly6G^hi^) were identified. The CD11b^hi^ cells (R4 region) were further gated into F4/80^-^ cells (R5) and F4/80^hi^ cells (R6). The F4/80+ cells (R6) were identified as mature MΦ and differentiating monocytes/inflammatory MΦ on the basis of higher expression of MHCII and Ly6C. The F4/80^-^ cells (R5) were gated for Ly6G and Ly6C expression to differentiate CD11b^hi^ Ly6G^hi^ Ly6C^int/low^ (PMN-MDSC) and CD11b^hi^ Ly6C^hi^ (MO-MDSC) as defined previously [19, 45, 46]. Further, multiple myeloid subsets were analyzed for expression of cell activation markers (e.g., CD80, CD86, MHCII, PD-L1 or CD115) to confirm the phenotype of each subset (S4 Fig). Since Ly6G is also expressed in mature neutrophils, we used alternate strategy to identify MDSC subsets by analyzing expression of CD49d, which is a Gr-1-independent marker differentiate mature neutrophils (Ly6G^+^) from PMN-MDSC (Ly6G^hi^ CD49d^-^) or MO-MDSC (Ly6C^hi^ CD49d^+^)for MDSC [46]. Specific cell populations are represented as a mean percentage of the cells from Ft-infected mice at various dpi in comparison to uninfected control mice (0 dpi). In addition to percentage of specific cell types observed, the total numbers of cells were calculated as well.

### Analysis for medullary and extra-medullary myelopoiesis

Bone marrow medullary myelopoiesis splenic extra-medullary myelopoiesis and progenitor-like cells were analyzed as described [41, 43]. Briefly, single cell suspensions were stained for lineage-positive markers (CD3ε, CD4, CD8α, CD11b, CD11c, CD19, B220, Gr-1, NK1.1, Ter119, TCRβ, γδTCR and FcεR1) and progenitor-like cell markers (c-kit, Sca-1, CD16, CD34 and CD150). Progenitor-like cells were quantified on the basis of Lin^−^/c-kit^+^/Sca-1^−^/CD34^+^ expressions.

### Myeloid cell isolation

Single cell suspension of lungs or spleen, mature myeloid cells and MDSC subsets (Ly6G and Ly6C fractions) were sorted using cell-specific antibody-coated magnetic beads (Miltenyi Biotec). The cell isolation scheme is shown in Supplementary procedure (S1 Procedures). Briefly, single-cell suspensions of lung or spleen were pelleted at 1200rpm for 5 min, incubated with biotinylated antibodies specific for lymphoid cells, alveolar macrophages, and mDC for depletion. From the negative fraction, sequential purification of Ly6G^+^ and Ly6C^+^ cells was performed. The sorted cells were confirmed as being >95% pure populations by flow cytometry and Giemsa staining of cytospin smears. For cytospin smear preparation, about 4 x10^4^ cells (in 200 μl final volume) was placed into the funnel attached to slide and spun at 1000rpm for 5 min. The smear was air-dried and stained using Hema-3 staining set (Protocol, Fischer Scientific).

### *In vitro* functional assays for MDSC

MDSC subsets were sorted as above. A small fraction of these cells (1 x 10^6^) were lysed in 0.05% SDS and serial dilutions were plated onto chocolate agar for counting cell-associated bacterial numbers. MDSC subsets and mature myeloid cells were tested for *in vitro* phagocytosis capacity. Briefly, sorted cells were initially cultured in DMEM for 4 h with Ft-LVS expressing GFP (Ft-GFP) at MOI = 200. After 4 and 24 h, cells were harvested and analyzed by flow cytometry to quantify cells harboring Ft-GFP. *In vitro* T cell proliferation assays were performed using plate-bound anti-CD3/CD28 antibodies. Briefly, MDSC subsets or mature MΦ sorted from the lungs and spleen of LVS-infected mice (3 and 6 dpi) were co-cultured with CFSE-labeled CD4^+^ T cells. As a control, mature Ly6C^+^ MΦ from naïve mice were used. On 6^th^ day, the extent of T cell proliferation was calculated by flow cytometric measurement of CFSE dilution. Maturation or differentiation of MDSC was tested *in vitro* by culturing the cells in presence or absence of rIFNγ (100U/ml medium), rTNFα (100ng/ml medium) or L-cell conditioned medium (1 ml medium for 5 x 10^5^ cells in 12 well-plates). The cultured cells were scored for maturation (on the basis of extent of monolayer confluence and cell morphology by phase-contrast microscopic examination) and live/dead cell counts (using 7-AAD staining) were evaluated at 24 or 48 h.

### Depletion of MDSC

For depletion of PMN- MDSC, Ft-infected mice were i.p injected with anti-Ly6G (clone 1A8) or isotype control rat IgG2a mAb (clone 2A3) antibody (200μg/mouse) daily from 2 to 4 dpi. The efficacy of MDSC depletion was monitored by myeloid cell counts from blood, lungs and spleen at 4 and 6 dpi using CD11b, CD11c, F4/80, Gr-1 (RB8-8C5) and Ly6C antibodies. In other experiments, mice were injected with anti-Gr-1 (clone RB6-8C5) or isotype control rat IgG2b mAb antibody (200μg/mouse) one day prior to the infection and again at 2 and 4 dpi. Cell depletion antibodies were purchased from BioXcell (Lebanon, NH). The efficacy of cell depletion was monitored by myeloid cell counts in lungs and spleen at 6 dpi using CD11b, CD11c, F4/80, Ly6G (1A8) and Ly6C antibodies. Antibody depleted mice were infected with Ft LVS (1000 or 500 cfu) and survival was monitored. As well, another group of mice was treated with 1A8 antibody one day prior to LVS infection (1000 cfu) and again at 2 and 4 dpi. Following infection, antibody depleted mice were monitored for survival or euthanized for cellular analysis on the indicated days.

### G-CSF neutralization

Ft LVS infected mice were systemically administered with anti-G-CSF antibody (mAB Rat IgG1, clone 67604) or its isotype control antibody (50μg/mouse) by retro-orbital vein injection on 1, 3 and 5 dpi post-infection. Following infection, antibody administered mice were monitored for survival or euthanized for cellular analysis on the indicated days. The mouse anti-G-CSF antibody was purchased from R&D system (Minneapolis, MN).

### GM-CSF treatment

Mice were treated with rGM-CSF (1 μg/mouse/daily once) either pre-infection at -7, -5, -3, and -1 dpi by intranasal administration or treated with rGM-CSF (2 μg/mouse/daily once) post-infection by intraperitoneal injection at 2 and 4 dpi. Following rGM-CSF treatment and infection, mice were monitored for survival or euthanized for cellular analysis on the indicated days. The rGM-CSF was purchased from Biolegend (San Diego, CA).

### Eliciting mature myeloid cell response, clodronate treatment, and lethal SchuS4 infection

To elicit mature myeloid cell responses, mice were infected with a sub-lethal (LD_50_) dose of LVS. At appropriate time points, mice were euthanized to study the cellular kinetics, bacterial burden, and tissue pathology. The survivors of sub-lethal infection were re-challenged with LVS (4000 cfu) or SchuS4 (20 or 150 cfu) at the end of 2, 4 or 8 weeks post-LVS sub-lethal infection. Some survivors of sub-lethal infected were also treated i.n. with clodronate-loaded or control liposomes (50 ul) at 21 and 23 days post-LVS infection, rested for 5 additional days, and then challenged with Ft SchuS4.

### Adoptive transfer of MDSC and LVS infection

Ly6G^+^ and Ly6C^+^ fractions of myeloid cells were isolated from CD45.1 donor mice at 6 day post-LVS infection as described above. Equal numbers of Ly6G^+^ (2 x 10^6^ cells) and Ly6C^+^ (2 x 10^6^ cells) fractions were mixed together in 2 ml of PBS and treated with ciprofloxacin (100μg/ml, final concentration) for 45 min to kill the cell-associated bacteria. As well, cells (2 x 10^6^) were treated with gentamicin (100μg/ml) for 45 min or left untreated with antibiotics for counting the intracellular bacterial burden. A small fraction of these cells (1 x 10^6^) were lysed in 0.05% SDS and serial dilutions were plated onto chocolate agar incubated at 37°C for 48 h and cfu numbers were calculated. Following antibiotic treatment, cells were washed twice with PBS to remove any residual antibiotics, prior to adoptive transfer into naïve mice. For adoptive transfer, cells (4 x 10^6^) were re-suspended in sterile PBS and aliquoted (50ul final volume) in hypodermic syringes for transfer. The cells were transferred to recipient CD45.2 mice by intra-tracheal route using a FiberOptic stylet and catheter tube (Kent Scientific, MA). Briefly, recipient mice were anesthetized with Ketamine (20mg/ml)/Xylazine (1mg/ml) mixture (80–100μl/mouse) and placed on a vertical support (glass stand), suspended by the incisors. Gently, the tongue was pulled out and held with thumb and forefinger. The middle finger supported the neck leaning on the glass stand. The fiberoptic cable with catheter tube was inserted through the vocal cords and light source guided the proper positioning of catheter in the trachea close to tracheal bifurcation. The fiberoptic cable was gently withdrawn carefully not to disturb the catheter. Cells were infused through the catheter while monitoring the mice for normal breathing. Later mice were placed in a supine position until recovery from anesthesia. Adoptive transfer of cells was evaluated by flow cytometry analysis of lung cells 1 day later. Recipient mice were left uninfected or infected next day with Ft LVS (1000 cfu) and survival was monitored. As a control, a group of naïve mice were infected with LVS alone. About 3–4 mice in each group were sacrificed on 6 dpi to assess lung pathology.

### Cell death analysis

Single cell suspensions from lungs or *in vitro* cultured MΦ were stained with 7-AAD, by TUNEL assay, or both, and then analyzed in an LSRII to enumerate dead cells. BAL fluid or culture supernatants were tested for LDH activity using the Cytotox96 non-radioactive kit (Promega).

### Statistical analysis

Statistical analysis and data compilation were done using GraphPad Prizm (ver 6). Student’s t-test or a parametric ANOVA test with Tukey’s post-test was used for statistical comparisons between groups. For survival analysis, Log-rank (Mantel-Cox) test was used. The p<0.05 was considered significant.

## Supporting Information

S1 Fig (Related to Fig 1) Exponential replication of live Ft, but not Ft LPS or inactivated Ft, activates chemokines/cytokines mediating acute inflammatory response. (A) Survival among males and females infected with Ft (% survival from two independent experiments). (B) Bacterial burden in liver and spleen in SchuS4 (10 cfu) or LVS (1000 cfu) infection (mean ± SD of two (SchuS4) or three (LVS) independent experiments). (C) Bacterial burden in tissues following 10^7^ cfu LVS infection (mean ± SD of two independent experiments). (D) Levels of eicanosoids and cytokines in lungs following SchuS4 (10 cfu) or LVS (1000 cfu) infection (mean ± SD of two (SchuS4) or three (LVS) independent experiments, * p<0.05). (E) Mortality pattern in Wt and *Casp1/11* ^-/-^ mice following sub-lethal LVS (500 cfu) infection (% survival of two independent experiments, Log-rank test, **p<0.01). (F) Survival following LPS (50 mg/kg) injection (% survival of two independent experiments). (G) Serum cytokine levels in LPS-injected mice at 24 h (mean ± SD of independent experiments, Student t-test, *p<0.05, **p<0.01). (H) Cytokine levels in supernatants of BMDM treated with LPS (100 ng/ml) for 24 h (mean ± SD of two independent experiments, Student t-test, *p<0.05, **p<0.01). (I) Lung cytokine levels in mice infected with live Ft LVS (10^7^ cfu) or inactivated-Ft (iFt) LVS (equivalent to 2 x10^7^ cfu) at 6 dpi (mean ± SD of two independent experiments, *p<0.05, **p<0.01). (J) Mean survival of mice infected with live (10^7^ cfu) or administered with iFt LVS (equivalent to 2 x10^7^ cfu) and lung pathology scores were low (4 ± 0.5) for iFt group versus LVS (14 ± 1.5).
(TIF)

S2 Fig (Related to Fig 2) Ft incites acute lung injury and necrotizing inflammation in vital organs. (A) The frequency of innate myeloid cells in Ft- infected lungs as determined by flow cytometry (mean ± SD of two (SchuS4, n = 6 mice) or three (LVS, n = 9 mice) independent experiments, Students t-test, *p<0.05, **p<0.01). (B) Lymphoid cell numbers in Ft- infected lungs as determined by flow cytometry (mean ± SD of two (SchuS4, n = 6 mice) or three (LVS, n = 9 mice) independent experiments, Students t-test, *p<0.05, **p<0.01). (C) Spleen weight and total cell numbers from control or LVS-infected mice (mean ± SD of two experiments, Student’s t-test, **p<0.05, *** p<0.001). (D) The cell numbers in BAL fluid collected from control or LVS-infected mice at 6 dpi (mean ± SD of two experiments, Student’s t-test, *** p<0.001). (E) SchuS4-infected lung shows lesions of acute lung injury such as diffused alveolar damage and hyaline membrane-like structure (arrows) lining the alveolar duct indicating at 1 and 3 dpi. Note thickened alveolar wall containing inflammatory exudate and clusters of gram-negative bacteria (long arrow) at 6 dpi. Images are representative of two independent experiments (HE, 400x). (F) Liver pathology in SchuS4-infected mice. Note inflammatory foci (arrow) and necrotic areas (nec) in the liver parenchyma (HE, 200x). (G) Histologic and immunohistochemical staining of lung tissues from Ft SchuS4 or LVS infected mice. (Top panel) Note microscopic images of lungs at 6 dpi show inflammatory foci with mixed cellular population like neutrophils, band cells (arrow), monocytes/macrophages and lymphocytes (HE, 400x). (Bottom panel) Note positive immunoreaction (brown color) for Ly6G+ cells in lung the sections of Ft-infected mice (IHC- counter-staining with Hematoxylin 7211, 400x). The microscopic images are representative of two independent experiments (n = 6 mice).
(TIF)

S3 Fig (Related to Fig 3) Ft alters medullary myelopoiesis in bone marrow and induces extra-medullary myelopoiesis in spleen. (A) Representative flow plots for analysis of progenitors-like cells in BM and spleen of LVS-infected mice. (B) Representative femur bones from Ft LVS-infected mice show grossly blanching of marrow indicating an altered myelopoiesis (C) Histology of control femur bone section shows cellular-rich bone marrow with normal erythroid (ep) and myeloid (mp) precursors, few megakaryocyte (arrow head), central vein (CV) and nutrient vein (nv). In contrast, femur bone section from LVS-infected (6 dpi) mice shows higher number of myeloid precursors (mp) and colony-forming structures (arrow) (HE, 400x). (D) Spleen histology shows enlarged white pulp (WP) and red pulp (RP) areas with increased myeloid precursors (arrow) and megakaryocytes indicating extra-medullary myelopoiesis (HE, 400x).
(TIF)

S4 Fig (Related to Fig 4) Multi-color flow cytometry for identification of myeloid cells in lungs. (A) Representative flow plots for a scheme of multiple myeloid cell analysis in lungs. Single cells from collagenase-treated lungs were stained for lymphoid marker (CD3, CD4, CD8, NK1.1, B220, CD19, Terr119 etc.), myeloid markers (CD11b, CD11c, F4/80, Gr-1, Ly6C, Ly6G etc.) and/or other markers (CD80, CD86, MHCII, PD-L1 or CD115). The lymphoid cells were excluded by dump gate and remaining cells (Region 1 or R1) were selected for further analysis. (B) R1 cells were gated for CD11b and CD11c expression to identify multiple subsets of myeloid cells (R2, R3 and R4 gates). (C) CD11b^+^CD11c^+^ cells (R3) were identified as either interstitial MΦ (F4/80^+^ Ly6C^+^MHCII^+^ Ly6G^-^), myeloid DC (F4/80^low/-^ Ly6C^low/-^ MHCII^+^Ly6G^-^) or PMN-DC hybrid (F4/80^-^ Ly6C^-^ MHCII^lo/-^Ly6G^hi^) subsets. (D) CD11c^hi^ (R2) cells were identified as alveolar MΦ (CD11b^-^CD11c^+^F4/80^+^ Ly6C^+^MHCII^+^ Ly6G^-^). (E) CD11b^hi^ cells (R4) were gated for F4/80 expression to distinguish immature myeloid cells from mature myeloid cells. (F) F4/80^+^ (R6) cells were identified as monocyte-differentiating inflammatory or mature MΦ (MHCII^+^Ly6C^+^LyG^-^). (G) The F4/80^-^ immature cells were further gated for expression of Ly6G and Ly6C expressions to identify Ly6G^hi^ Ly6C^int/low^ or Ly6C^hi^ Ly6G^-^. These cells were identified as PMN-MDSC (Ly6G^hi^ Ly6C^int/low^) and MO-MDSC (Ly6C^hi^ Ly6G^-^) based on the level of expression of CD115/PD-L1/Arg-1 or MHCII/CD80/CD86 markers. In histogram, the vertical line represents the cut-off for defining the positive populations based on the level of expression of indicated marker in control cells. (H) The frequency of multiple myeloid cell subsets in lungs (mean ± SD of two (SchuS4, n = 6 mice) or three (LVS, n = 9 mice) independent experiments, Students t-test, *p<0.05). (I) Geimsa stained cytospin smears of BAL fluid showing a large number of band cells (immature) and poly-morphonuclear cells (neutrophils). Note the majority of cells are morphologically consistent with alveolar macrophages and other mononuclear cells in control mouse.
(TIF)

S5 Fig (Related to Fig 5) Functional characterization of IMC/MDSC in pulmonary tularemia. (A) Representative flow plots for *in vitro* phagocytic assay with naïve BMDM (as positive control), Ly6G^+^ (PMN-MDSC) or Ly6C^+^ (MO-MDSC) cells. (B) Representative histogram of CFSE dilution for *in vitro* T cell proliferation assay with Ly6G^+^ (PMN-MDSC) or Ly6C^+^ (MO-MDSC) cells as accessory cells. (C) Representative phase-contrast microscopic images of *in vitro* maturation/differentiation assay for BMDM (positive control) or Ly6C^+^ (MO-MDSC) cells.
(TIF)

S6 Fig (Related to Fig 6) Predominance of IMC/MDSC fails to protect mice from lethal pulmonary tularemia. (A) Frequency of Gr-1^+^ cells in Ft LVS-infected mice treated with 1A8 antibody (mean ± SD of two independent experiments, Student’s t-test **p<0.01). (B) Frequency and numbers of Ly6G^+^ or Ly6C^+^ cells in Ft LVS-infected mice treated with RB6-8C5 antibody (mean ± SD of two independent experiments, Student’s t-test *p<0.05, **p<0.01). (C) Ratio of immature myeloid cells (IMC) *versus* mature myeloid cells (MMC) in bone marrow (BM) and lungs with and without anti-G-CSF antibody treatment in LVS (1000 cfu) infected mice (mean ± SD, n = 3–5 mice, Student’s t-test, *p<0.05). (D) Survival following anti-G-CSF antibody treatment in LVS (1000 cfu) infected mice (% survival, n = 6/group). (E) Tissue bacterial burden in mice infected with sub-lethal (LD_50_) LVS at various days post-infection (mean ± SD from two independent experiments, Student’s t-test, *p<0.05). (F) Numbers of lymphoid cells in lungs of sub-lethally LVS-infected survivor mice (mean ± SD of two independent experiments, Student’s t-test, *p<0.05, **p<0.01). (G) Tissue bacterial burden in mice infected with sub-lethal (LD_50_) LVS at various weeks post-infection (mean ± SD, n = 3–4 mice). (H) Numbers of myeloid cells in lungs of LVS-infected mice treated with rGM-CSF at pre-infection or post-infection (mean ± SD of 4 mice, Student’s t-test, *p<0.05). (I) Bacterial burden in lungs of LVS-infected mice treated with rGM-CSF at pre-infection or post-infection (mean ± SD of 4 mice). (J) Lung pathology score in mice adoptively transferred with and without Ly6G/C cells followed by LVS infection (mean ± SD of 3 mice, Student’s t-test, *p<0.05). (K) Representative microscopic images of lung pathology in mice adoptively transferred with and without Ly6G/C cells followed by LVS infection.
(TIF)

S1 Procedures a) Histopathology scoring criteria for microscopic lesions observed in Ft-infected tissues. b) Scheme of myeloid cell subsets isolation by magnetic antibody beads.
(DOCX)
