# Supplementary figures and images for: An Immature Myeloid/Myeloid-Suppressor Cell Response Associated with Necrotizing Inflammation Mediates Lethal Pulmonary Tularemia
Source: PLoS Pathog. 2016 Mar 25;12(3):e1005517. doi: 10.1371/journal.ppat.1005517 (PMC4807818; doi:10.1371/journal.ppat.1005517)

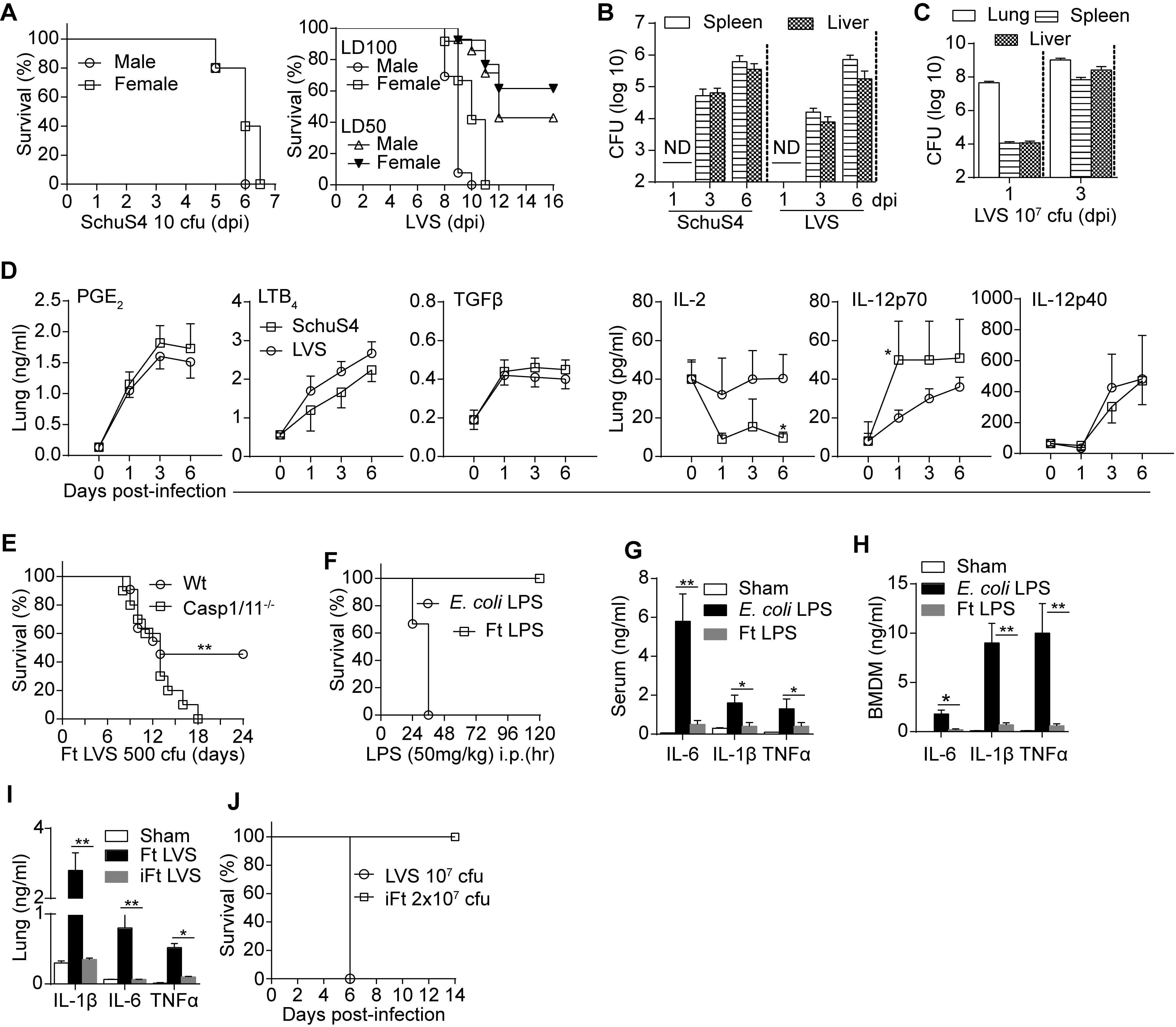

Supplement: S1 Fig — (A) Survival among males and females infected with Ft (% survival from two independent experiments). (B) Bacterial burden in liver and spleen in SchuS4 (10 cfu) or LVS (1000 cfu) infection (mean ± SD of two (SchuS4) or three (LVS) independent experiments). (C) Bacterial burden in tissues following 107 cfu LVS infection (mean ± SD of two independent experiments). (D) Levels of eicanosoids and cytokines in lungs following SchuS4 (10 cfu) or LVS (1000 cfu) infection (mean ± SD of two (SchuS4) or three (LVS) independent experiments, * p<0.05). (E) Mortality pattern in Wt and Casp1/11 -/- mice following sub-lethal LVS (500 cfu) infection (% survival of two independent experiments, Log-rank test, **p<0.01). (F) Survival following LPS (50 mg/kg) injection (% survival of two independent experiments). (G) Serum cytokine levels in LPS-injected mice at 24 h (mean ± SD of independent experiments, Student t-test, *p<0.05, **p<0.01). (H) Cytokine levels in supernatants of BMDM treated with LPS (100 ng/ml) for 24 h (mean ± SD of two independent experiments, Student t-test, *p<0.05, **p<0.01). (I) Lung cytokine levels in mice infected with live Ft LVS (107 cfu) or inactivated-Ft (iFt) LVS (equivalent to 2 x10^7^ cfu) at 6 dpi (mean ± SD of two independent experiments, *p<0.05, **p<0.01). (J) Mean survival of mice infected with live (107 cfu) or administered with iFt LVS (equivalent to 2 x10^7^ cfu) and lung pathology scores were low (4 ± 0.5) for iFt group versus LVS (14 ± 1.5). (TIF) [file ppat.1005517.s001.tif]

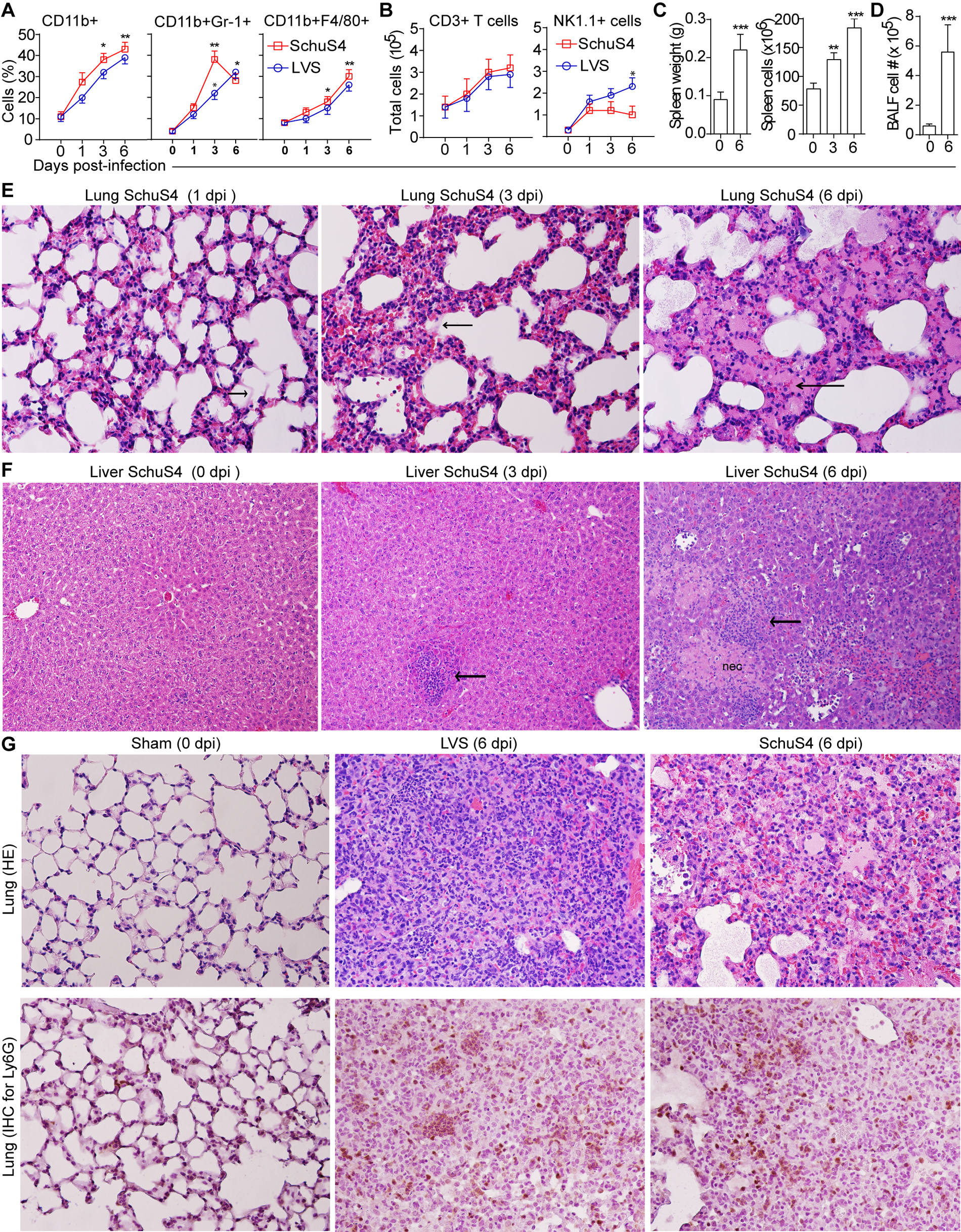

Supplement: S2 Fig — (A) The frequency of innate myeloid cells in Ft- infected lungs as determined by flow cytometry (mean ± SD of two (SchuS4, n = 6 mice) or three (LVS, n = 9 mice) independent experiments, Students t-test, *p<0.05, **p<0.01). (B) Lymphoid cell numbers in Ft- infected lungs as determined by flow cytometry (mean ± SD of two (SchuS4, n = 6 mice) or three (LVS, n = 9 mice) independent experiments, Students t-test, *p<0.05, **p<0.01). (C) Spleen weight and total cell numbers from control or LVS-infected mice (mean ± SD of two experiments, Student’s t-test, **p<0.05, *** p<0.001). (D) The cell numbers in BAL fluid collected from control or LVS-infected mice at 6 dpi (mean ± SD of two experiments, Student’s t-test, *** p<0.001). (E) SchuS4-infected lung shows lesions of acute lung injury such as diffused alveolar damage and hyaline membrane-like structure (arrows) lining the alveolar duct indicating at 1 and 3 dpi. Note thickened alveolar wall containing inflammatory exudate and clusters of gram-negative bacteria (long arrow) at 6 dpi. Images are representative of two independent experiments (HE, 400x). (F) Liver pathology in SchuS4-infected mice. Note inflammatory foci (arrow) and necrotic areas (nec) in the liver parenchyma (HE, 200x). (G) Histologic and immunohistochemical staining of lung tissues from Ft SchuS4 or LVS infected mice. (Top panel) Note microscopic images of lungs at 6 dpi show inflammatory foci with mixed cellular population like neutrophils, band cells (arrow), monocytes/macrophages and lymphocytes (HE, 400x). (Bottom panel) Note positive immunoreaction (brown color) for Ly6G+ cells in lung the sections of Ft-infected mice (IHC- counter-staining with Hematoxylin 7211, 400x). The microscopic images are representative of two independent experiments (n = 6 mice). (TIF) [file ppat.1005517.s002.tif]

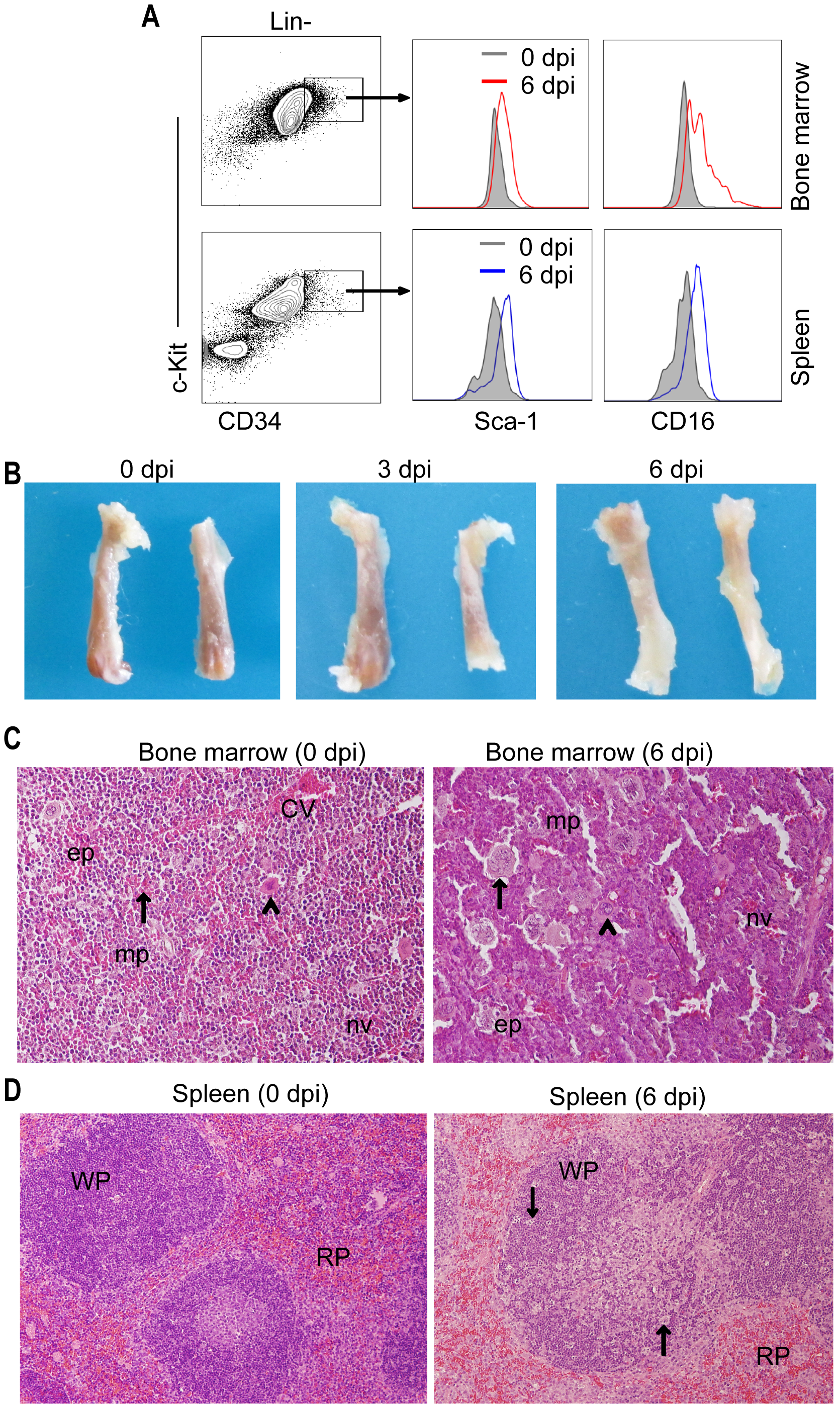

Supplement: S3 Fig — (A) Representative flow plots for analysis of progenitors-like cells in BM and spleen of LVS-infected mice. (B) Representative femur bones from Ft LVS-infected mice show grossly blanching of marrow indicating an altered myelopoiesis (C) Histology of control femur bone section shows cellular-rich bone marrow with normal erythroid (ep) and myeloid (mp) precursors, few megakaryocyte (arrow head), central vein (CV) and nutrient vein (nv). In contrast, femur bone section from LVS-infected (6 dpi) mice shows higher number of myeloid precursors (mp) and colony-forming structures (arrow) (HE, 400x). (D) Spleen histology shows enlarged white pulp (WP) and red pulp (RP) areas with increased myeloid precursors (arrow) and megakaryocytes indicating extra-medullary myelopoiesis (HE, 400x). (TIF) [file ppat.1005517.s003.tif]

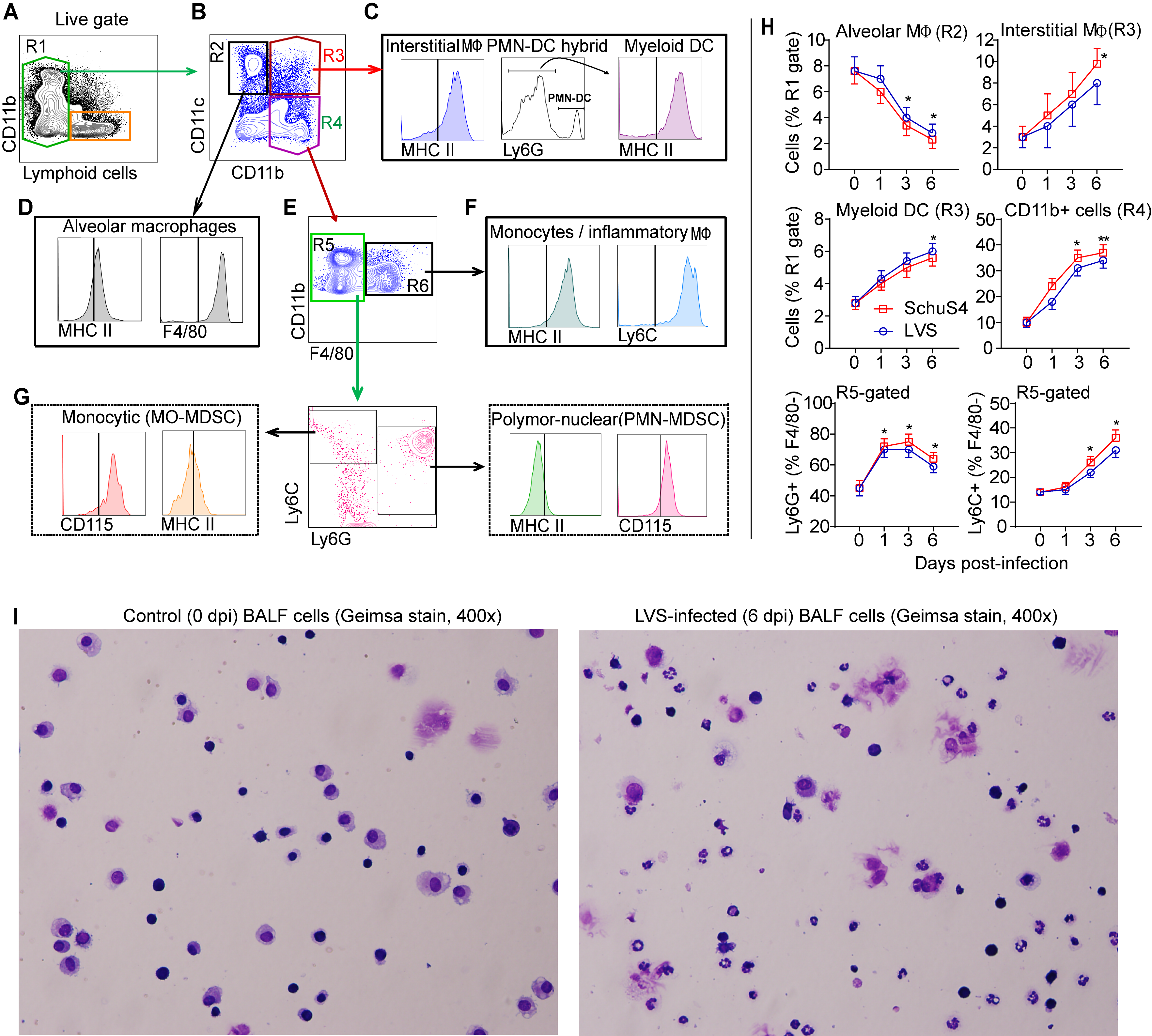

Supplement: S4 Fig — (A) Representative flow plots for a scheme of multiple myeloid cell analysis in lungs. Single cells from collagenase-treated lungs were stained for lymphoid marker (CD3, CD4, CD8, NK1.1, B220, CD19, Terr119 etc.), myeloid markers (CD11b, CD11c, F4/80, Gr-1, Ly6C, Ly6G etc.) and/or other markers (CD80, CD86, MHCII, PD-L1 or CD115). The lymphoid cells were excluded by dump gate and remaining cells (Region 1 or R1) were selected for further analysis. (B) R1 cells were gated for CD11b and CD11c expression to identify multiple subsets of myeloid cells (R2, R3 and R4 gates). (C) CD11b+CD11c+ cells (R3) were identified as either interstitial MΦ (F4/80+ Ly6C+MHCII+ Ly6G-), myeloid DC (F4/80low/- Ly6Clow/- MHCII+Ly6G-) or PMN-DC hybrid (F4/80- Ly6C- MHCIIlo/-Ly6Ghi) subsets. (D) CD11chi (R2) cells were identified as alveolar MΦ (CD11b-CD11c+F4/80+ Ly6C+MHCII+ Ly6G-). (E) CD11bhi cells (R4) were gated for F4/80 expression to distinguish immature myeloid cells from mature myeloid cells. (F) F4/80+ (R6) cells were identified as monocyte-differentiating inflammatory or mature MΦ (MHCII+Ly6C+LyG-). (G) The F4/80- immature cells were further gated for expression of Ly6G and Ly6C expressions to identify Ly6Ghi Ly6Cint/low or Ly6Chi Ly6G-. These cells were identified as PMN-MDSC (Ly6Ghi Ly6Cint/low) and MO-MDSC (Ly6Chi Ly6G-) based on the level of expression of CD115/PD-L1/Arg-1 or MHCII/CD80/CD86 markers. In histogram, the vertical line represents the cut-off for defining the positive populations based on the level of expression of indicated marker in control cells. (H) The frequency of multiple myeloid cell subsets in lungs (mean ± SD of two (SchuS4, n = 6 mice) or three (LVS, n = 9 mice) independent experiments, Students t-test, *p<0.05). (I) Geimsa stained cytospin smears of BAL fluid showing a large number of band cells (immature) and poly-morphonuclear cells (neutrophils). Note the majority of cells are morphologically consistent with alveolar macrophages and other mononuclear [file ppat.1005517.s004.tif]

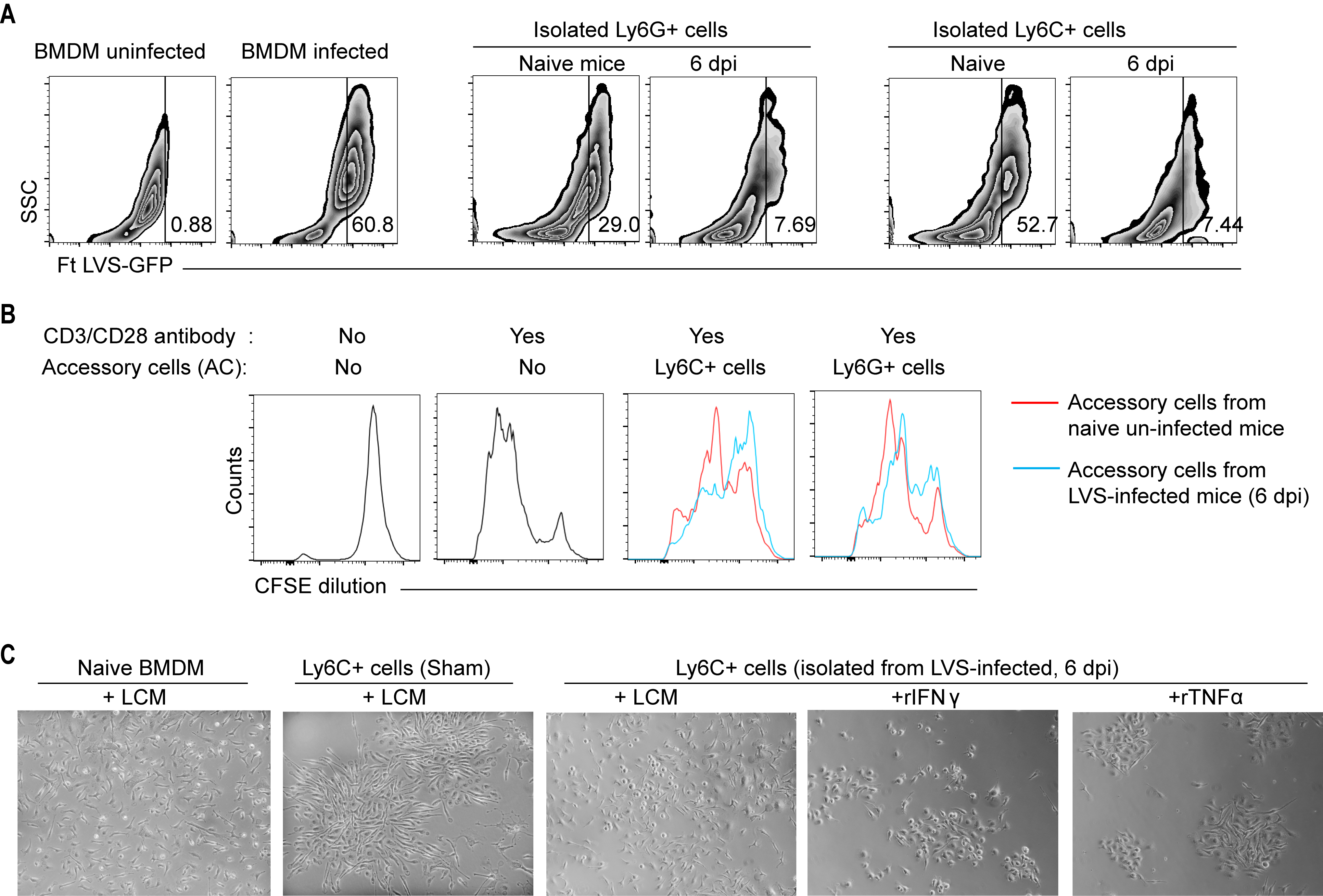

Supplement: S5 Fig — (A) Representative flow plots for in vitro phagocytic assay with naïve BMDM (as positive control), Ly6G+ (PMN-MDSC) or Ly6C+ (MO-MDSC) cells. (B) Representative histogram of CFSE dilution for in vitro T cell proliferation assay with Ly6G+ (PMN-MDSC) or Ly6C+ (MO-MDSC) cells as accessory cells. (C) Representative phase-contrast microscopic images of in vitro maturation/differentiation assay for BMDM (positive control) or Ly6C+ (MO-MDSC) cells. (TIF) [file ppat.1005517.s005.tif]

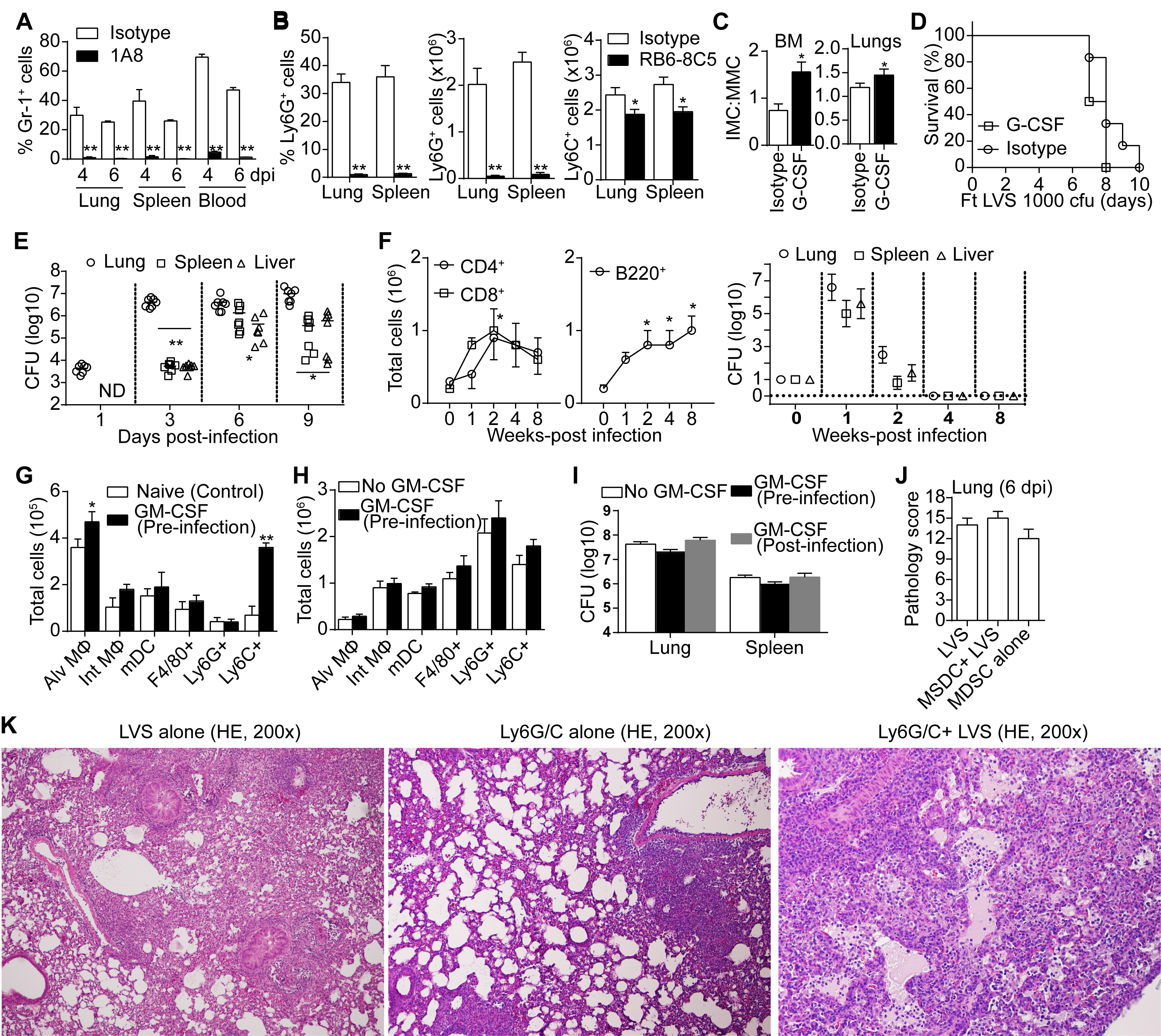

Supplement: S6 Fig — (A) Frequency of Gr-1+ cells in Ft LVS-infected mice treated with 1A8 antibody (mean ± SD of two independent experiments, Student’s t-test **p<0.01). (B) Frequency and numbers of Ly6G+ or Ly6C+ cells in Ft LVS-infected mice treated with RB6-8C5 antibody (mean ± SD of two independent experiments, Student’s t-test *p<0.05, **p<0.01). (C) Ratio of immature myeloid cells (IMC) versus mature myeloid cells (MMC) in bone marrow (BM) and lungs with and without anti-G-CSF antibody treatment in LVS (1000 cfu) infected mice (mean ± SD, n = 3–5 mice, Student’s t-test, *p<0.05). (D) Survival following anti-G-CSF antibody treatment in LVS (1000 cfu) infected mice (% survival, n = 6/group). (E) Tissue bacterial burden in mice infected with sub-lethal (LD50) LVS at various days post-infection (mean ± SD from two independent experiments, Student’s t-test, *p<0.05). (F) Numbers of lymphoid cells in lungs of sub-lethally LVS-infected survivor mice (mean ± SD of two independent experiments, Student’s t-test, *p<0.05, **p<0.01). (G) Tissue bacterial burden in mice infected with sub-lethal (LD50) LVS at various weeks post-infection (mean ± SD, n = 3–4 mice). (H) Numbers of myeloid cells in lungs of LVS-infected mice treated with rGM-CSF at pre-infection or post-infection (mean ± SD of 4 mice, Student’s t-test, *p<0.05). (I) Bacterial burden in lungs of LVS-infected mice treated with rGM-CSF at pre-infection or post-infection (mean ± SD of 4 mice). (J) Lung pathology score in mice adoptively transferred with and without Ly6G/C cells followed by LVS infection (mean ± SD of 3 mice, Student’s t-test, *p<0.05). (K) Representative microscopic images of lung pathology in mice adoptively transferred with and without Ly6G/C cells followed by LVS infection. (TIF) [file ppat.1005517.s006.tif]
