# Supplementary material for: An Immature Myeloid/Myeloid-Suppressor Cell Response Associated with Necrotizing Inflammation Mediates Lethal Pulmonary Tularemia
Source: PLoS Pathog. 2016 Mar 25;12(3):e1005517. doi: 10.1371/journal.ppat.1005517 (PMC4807818; doi:10.1371/journal.ppat.1005517)
Supplement: S1 Procedures — a) Histopathology scoring criteria for microscopic lesions observed in Ft-infected tissues. b) Scheme of myeloid cell subsets isolation by magnetic antibody beads. (DOCX) [file ppat.1005517.s007.docx]

**S1 Procedures:**

1. **Histopathology scoring criteria for microscopic lesions observed in Ft-infected tissues:** HE stained sections of lungs, spleen and liver obtained at various dpi were evaluated for type and extent of inflammatory changes. The lesions were semi-quantitatively scored using criteria as shown below.

| **Organ and changes** | **Score** |
| --- | --- |
| Lungs  1) No lesions  2) Location of inflammation  a) Peribronchiolar or perivascular  b) Alveolar lumen and alveolar wall  c) a + b  d) a+ b and exudates in bronchi/bronchiolar lumen  3) Type of cellular infiltrates in the inflammatory foci  a) Neutrophils (a few)  b) Neutrophils (predominant) and monocytes/macrophages  b) Neutrophils and macrophages and lymphocytes (mixed infiltrates)  c) Mixed infiltrates and granulomatous foci,  4) Extent of inflammation in the lung parenchyma  a) Patchy (small) inflammatory foci, few  b) Patchy inflammatory foci, many  c) Large inflammatory foci, many  d) Necrotizing inflammatory foci  5) Extent of necrotic changes  a) Small necrotic foci, few  b) Small necrotic foci, many  c) Large necrotic foci | 0  1  2  3  4  1  2  3  4  1  2  3  4  2  3  4 |
| Spleen  a) No lesions  a) Splenomegaly  b) Marginal zone thickening and red pulp inflammation  c) Granulomatous inflammation | 0  2  3  4 |
| Liver  a) No lesions  b) Hepatic lobular infiltration by neutrophils and mononuclear cells  b) Granulomatous inflammation  c) Necrotizing and granulomatous inflammation | 0  2  3  4 |

1. **Scheme of myeloid cell subsets isolation by using magnetic antibody beads**: Since live cells from Ft SchuS4 or LVS infected lung/spleen could not be processed through FACS Aria sorter (due to biosecurity measures), we followed the magnetic sorting for isolation of myeloid cells from Ft-infected mice (as shown below). In addition, due to magnetic sorting, we were unable to exclude the dead cells from different fractions of isolated cells. The isolated subsets were confirmed for the purity and efficiency of magnetic separation by staining with subset-specific surface markers.

Single cell suspension from lungs or spleen

Add biotinylated CD3/CD4/CD8/NK 1.1/γδ TCR/CD19/B220/Terr119/F480 and CD11c antibodies

(15 min incubation)

Add anti-biotin microbeads (Miltenyi) for 15 min

Negative fraction T cells/B cells/NK cells/DC are depleted

Add PE-Ly6G ab (20 min)

Add Anti-PE microbeads (20 min)

Positive selection of CD11b^+^Ly6G^+^ cells Negative fraction

Add FITC-Ly6C Ab (15’)

Add Anti-FITC microbeads (20 min)

Positive selection of CD11b+Ly6C+ cells Discard negative fraction
